# RGD peptide in cancer targeting: Benefits, challenges, solutions, and possible integrin–RGD interactions

**DOI:** 10.1002/cam4.6800

**Published:** 2024-02-01

**Authors:** Hossein Javid, Mahsa Akbari Oryani, Nastaran Rezagholinejad, Ali Esparham, Mahboubeh Tajaldini, Mehdi Karimi‐Shahri

**Affiliations:** ^1^ Department of Medical Laboratory Sciences Varastegan Institute for Medical Sciences Mashhad Iran; ^2^ Department of Clinical Biochemistry, Faculty of Medicine Mashhad University of Medical Sciences Mashhad Iran; ^3^ Surgical Oncology Research Center Mashhad University of Medical Sciences Mashhad Iran; ^4^ Department of Pathology, School of Medicine Mashhad University of Medical Sciences Mashhad Iran; ^5^ Department of Biochemistry Payame Noor University (PNU) Mashhad Iran; ^6^ Student Research Committee, Faculty of Medicine Mashhad University of Medical Sciences Mashhad Iran; ^7^ Ischemic Disorder Research Center Golestan University of Medical Sciences Gorgan Iran; ^8^ Department of Pathology, School of Medicine Gonabad University of Medical Sciences Gonabad Iran

**Keywords:** cancer targeting, challenges, conjugation process, RGD peptides

## Abstract

RGD peptide can be found in cell adhesion and signaling proteins, such as fibronectin, vitronectin, and fibrinogen. RGD peptides' principal function is to facilitate cell adhesion by interacting with integrin receptors on the cell surface. They have been intensively researched for use in biotechnology and medicine, including incorporation into biomaterials, conjugation to medicinal molecules or nanoparticles, and labeling with imaging agents. RGD peptides can be utilized to specifically target cancer cells and the tumor vasculature by engaging with these integrins, improving drug delivery efficiency and minimizing adverse effects on healthy tissues. RGD‐functionalized drug carriers are a viable option for cancer therapy as this focused approach has demonstrated promise in the future. Writing a review on the RGD peptide can significantly influence how drugs are developed in the future by improving our understanding of the peptide, finding knowledge gaps, fostering innovation, and making drug design easier.

## 1 INTRODUCTION

**FIGURE 1:**
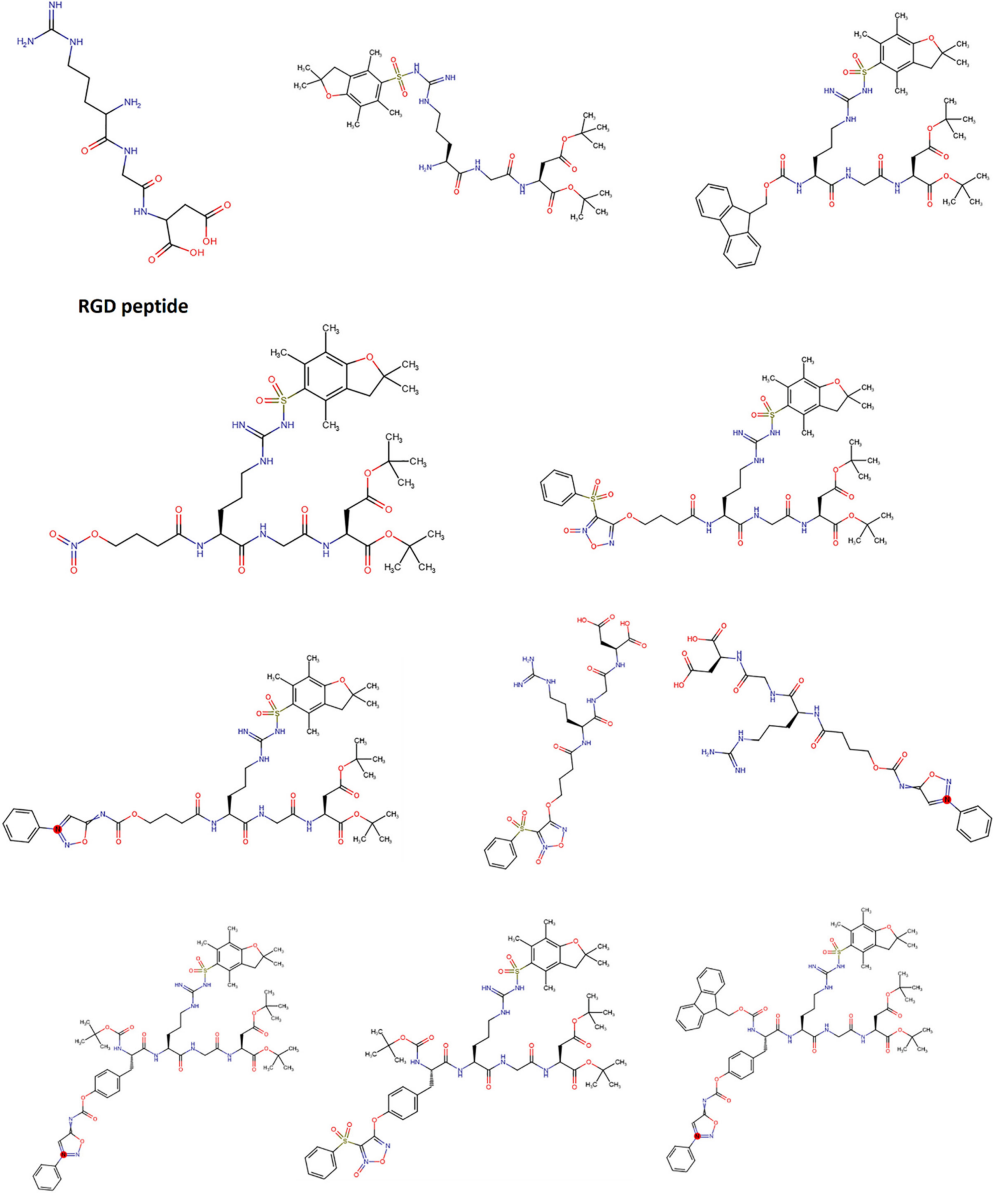
Examples of RGD analouges.

Three amino acids compose the structure of the RGD peptide: arginine (Arg), glycine (Gly), and aspartic acid (Asp). The RGD motif is formed by a linear arrangement of these amino acids.^1^, ^2^ This peptide can be found in cell adhesion and signaling proteins, such as fibronectin, vitronectin, and fibrinogen.^3^, ^4^, ^5^ RGD peptides' principal role is to facilitate cell adhesion by binding to the integrin receptors on the cell's surface.^1^ Integrins are proteins located in the transmembrane that are essential for cell signaling, motility, and survival. They participate in a variety of biological processes, such as immune response, wound healing, and angiogenesis.^5^, ^6^, ^7^, ^8^ In the timeline of RGD peptide, the RGD sequence was first identified as a crucial motif for cell adhesion in the extracellular matrix in 1984. In the 1990s, the RGD peptide began to be modified by scientists to enhance its binding affinity and selectivity to specific integrin receptors. By 1997, biomaterials were incorporated with RGD peptide for tissue engineering, promoting cell adhesion and tissue regeneration. In the early 2000s, the use of RGD peptide as a targeting ligand for drug delivery systems was explored by researchers, enabling specific delivery to cells expressing integrin receptors. In 2010, RGD peptide‐conjugated nanoparticles were developed for targeted delivery of anticancer drugs to tumor cells, showing promising results in preclinical studies. Since 2015, RGD peptide has also been utilized in the development of scaffolds and hydrogels for tissue engineering applications, facilitating cell adhesion and promoting tissue regeneration. In 2020, RGD peptide was incorporated into bioinks used in 3D bioprinting, enabling precise deposition of cells and promoting their attachment to the printed structures. They found that these integrins bind to their native ligands that contain the RGD sequence. This discovery opened up new possibilities for utilizing RGD peptides as targeting motifs in cancer treatment for both therapeutic and diagnostic purposes. The absence of intellectual property protection further fueled the development of various RGD‐based agents, including fluorescence markers, radiopharmaceuticals, drug conjugates, nanoparticles, and micelles. While RGD applications related to angiogenesis and αvβ3‐integrin have been prominent in the field of radiopharmacy, it is important to note that RGD is not limited to these specific targets. Researchers have also explored the affinity and selectivity of RGD peptides for other integrins, such as αvβ6‐integrin. The development of radiolabeled ligands targeting αvβ3‐integrin has been a significant breakthrough in radiotheranostics for cancer therapy. The future of RGD in targeted cancer therapy holds great promise. Ongoing research is focused on the continuous development of novel RGD‐based ligands and exploring the potential of integrins in therapy. RGD peptides may find applications beyond cancer therapy, including in the field of cancer immunotherapy. The complexity of integrin functions suggests that future research on RGD‐related molecules will encompass diverse areas of molecular medicine and life science.^9^, ^10^, ^11^, ^12^, ^13^, ^14^, ^15^ RGD peptides can be modified to enhance its anticancer properties. A few Examples of RGD analogues that were used for the targeting cancer were depicted in Figure 1.

Specific integrins identify the RGD motif within proteins and bind to this peptide, allowing cell adhesion and communication. RGD peptides have been widely explored for diverse uses in medicine and biotechnology due to their ability to improve cell adhesion and target particular integrin receptors.^10^, ^16^, ^17^, ^18^, ^19^ To increase cell adhesion and growth, RGD peptides can be introduced into biomaterials such as hydrogels and scaffolds. This may enhance these materials' biocompatibility and efficacy in the regeneration and repair of tissues.^20^, ^21^, ^22^, ^23^

**FIGURE 2:**
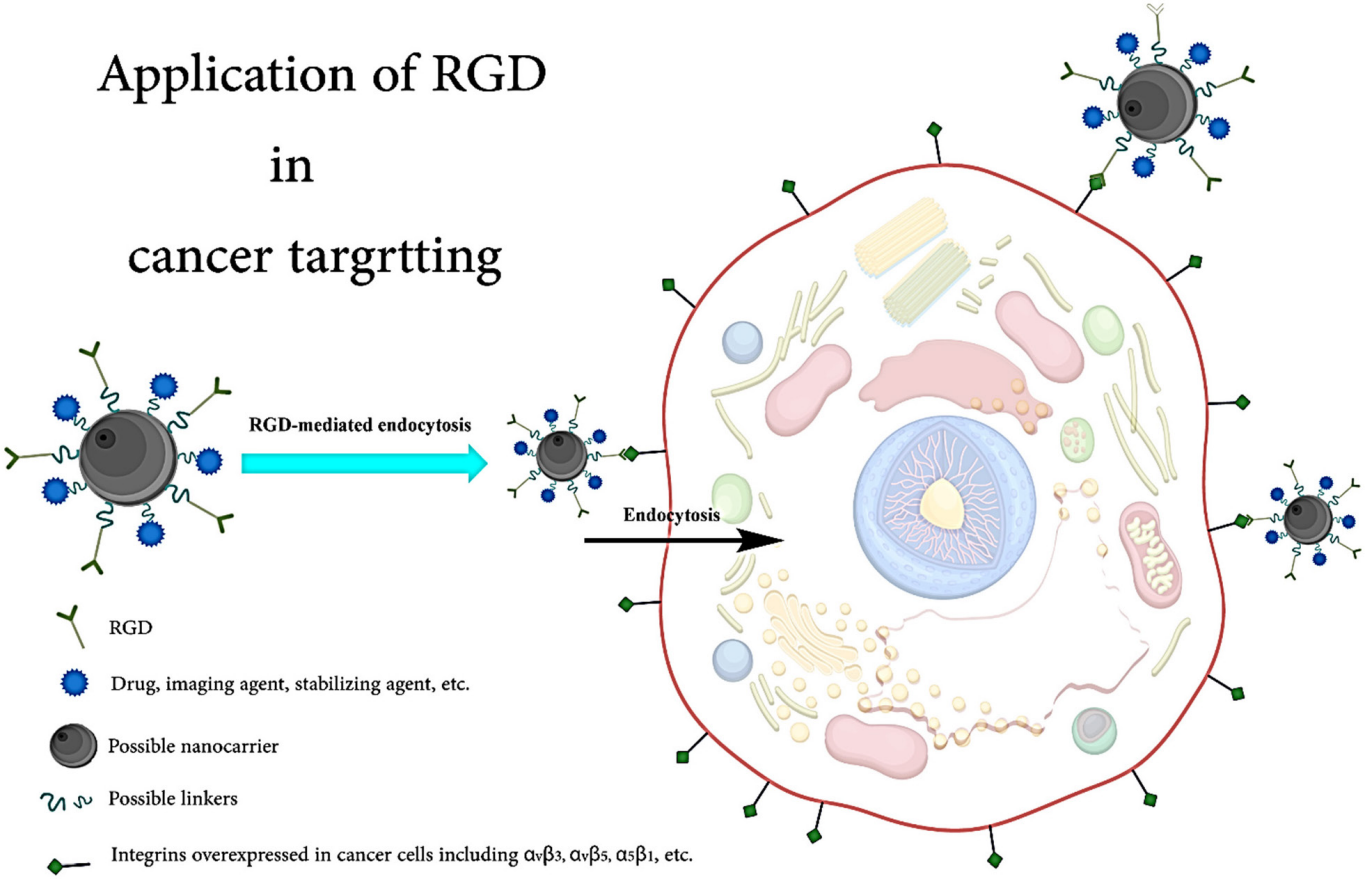
Receptor‐mediated endocytosis using RGD‐peptide drug, or carrier, etc. conjugates.

To target particular integrin receptors that are overexpressed in illnesses such as cancer, RGD peptides can be coupled to therapeutic molecules or nanoparticles.^5^, ^24^, ^25^, ^26^, ^27^ Integrins, notably αvβ3 and αvβ5, are elevated in malignant cells and vasculature. The medicine may be administered selectively to the tumor location by connecting RGD peptides to drug carriers, limiting the impact on normal cells and tissues. RGD peptides can help drug carriers get into cells by engaging with integrin receptors, which are found on the cell's surface. This receptor‐dependent endocytosis can boost the absorption of drugs in the cell, resulting in greater therapeutic effectiveness (Figure 2).^28^, ^29^, ^30^, ^31^ This is especially effective for delivering drugs with low cell permeability or that breakdown quickly in the extracellular environment. RGD‐functionalized drug carriers can be engineered to carry numerous therapeutic agents to the target area, such as chemotherapeutic medicines and genes. This can aid in the treatment of drug resistance and improve the overall therapeutic impact.

RGD‐functionalized drug carriers can be programmed to release their payload in response to external stimuli like as pH, temperature, or the presence of certain enzymes.^32^, ^33^, ^34^ This can guarantee that the medicine is only delivered at the intended spot, reducing adverse effects, and boosting therapeutic efficacy. To observe integrin expression in animal models, RGD peptides can be tagged with imaging agents like fluorescent dyes or radiotracers.^35^, ^36^, ^37^ This can aid in identifying and monitoring illnesses caused by aberrant integrin expressions, such as cancer and cardiovascular disease. Furthermore, RGD peptides can be combined with imaging agents to allow for real‐time monitoring of drug distribution and treatment response. This can aid in the optimization of treatment procedures and give crucial information on the efficacy of the therapy.^38^, ^39^, ^40^, ^41^, ^42^

By inhibiting the interaction between integrins and their ligands, RGD peptides can be utilized alone to limit tumor development and angiogenesis. This has the potential to alter signaling pathways involved in cancer cell survival, migration, and invasion. Angiogenesis inhibition is a viable treatment option for cancer and other disorders characterized by excessive blood vessel formation. RGD peptide‐conjugated medicines can preferentially target angiogenesis‐related integrins, resulting in more effective anti‐angiogenic therapy.^43^, ^44^, ^45^, ^46^

RGD peptides, RGD peptide‐conjugated medicines, and RGD peptide‐conjugated nanoparticles/nanocarriers have considerable potential for targeted drug delivery and imaging. Their capacity to selectively target integrin‐expressing cells may result in better treatment results, fewer side effects, and enhanced disease identification and monitoring. New medications targeting the RGD peptide have the potential to enhance the lives of millions of individuals suffering from illnesses such as cancer, cardiovascular disease, and inflammatory ailments. A thorough review can aid in the consolidation of existing information regarding RGD peptides, their interactions with integrins, and their significance in many biological processes. This can serve as a good basis for researchers to find new therapeutic targets. A review of RGD peptides can have a substantial influence on future drug development by improving our understanding of the peptide, revealing knowledge gaps, promoting innovation, and aiding drug design.

## 2 OVEREXPRESSED INTEGRINS FOR RGD PEPTIDE‐BASED CANCER THERAPIES

A class of cell surface receptors known as integrins is essential for cell adhesion, migration, and signaling. They are heterodimeric proteins composed of two subunits, α and β, which come together to create different integrin pairs. Certain integrins are particularly appealing targets for RGD peptide‐based therapeutics because they are overexpressed on the surfaces of cancer cells and the tumor vasculature in malignancies.^15^, ^47^, ^48^, ^49^ Integrins that recognize the RGD motif particularly interact with the RGD peptide. Several cancer cell types, including melanoma, glioblastoma, and breast, prostate, and ovarian malignancies, overexpress the αvβ3 integrin.^50^, ^51^, ^52^, ^53^, ^54^ Additionally, it supports angiogenesis by being abundantly expressed in the tumor vasculature.^55^, ^56^, ^57^ RGD peptides that target the αvβ3 integrin can aid in preventing tumor angiogenesis, invasion, and proliferation. Similar to αvβ3, αvβ5 integrin is overexpressed in a variety of cancer types and contributes to tumor angiogenesis.^58^, ^59^, ^60^, ^61^ To stop the creation of new blood vessels and restrain tumor growth, RGD peptides can interfere with this integrin's function. α5β1 integrin, which is overexpressed in several malignancies including breast, lung, and colon cancers, is involved in cell adhesion and migration.^62^, ^63^, ^64^, ^65^ RGD peptides can help stop cancer cells from migrating and invading by targeting the α5β1 integrin. Numerous malignancies, such as pancreatic, lung, and colon tumors, have elevated levels of the integrin αvβ6.^36^, ^66^, ^67^, ^68^ Cancer cell invasion and metastasis include the αvβ6 integrin. It is possible to stop the spread of cancer by targeting this integrin using RGD peptides. The surface of platelets expresses the αIIbβ3 integrin, which is essential for platelet aggregation. Even while platelet aggregation is not directly connected to tumor cells, tumor cells can use it to encourage metastasis. Inhibiting platelet aggregation and lowering the risk of metastasis can be accomplished by targeting αIIbβ3 integrin with RGD peptides.^69^, ^70^, ^71^, ^72^ RGD peptides can be utilized to specifically target cancer cells and the tumor vasculature by engaging with these integrins, improving drug delivery efficiency, and minimizing adverse effects on healthy tissues. RGD‐functionalized drug carriers are a viable option for cancer therapy since this focused approach has demonstrated promise in studies.

## 3 RGD PEPTIDES: CHALLENGES

The potential applications of the RGD peptide in cancer targeting have garnered significant attention within the field of cancer research. The discovery of the RGD peptide's cancer targeting properties has opened up novel avenues for the precise delivery of drugs and imaging agents in cancer therapy. By linking anticancer drugs or imaging agents to the RGD peptide, cancer cells can be specifically targeted while minimizing harm to healthy cells. This targeted approach holds promise for improving the effectiveness of cancer treatments and reducing undesirable side effects. Additionally, the RGD peptide can be employed in the development of imaging agents that are specific to cancer. By attaching a radioactive or fluorescent label to the RGD peptide, it becomes feasible to visualize and detect tumors using diverse imaging techniques such as positron emission tomography (PET) or fluorescence imaging.^9^, ^10^, ^15^, ^25^, ^73^, ^74^, ^75^, ^76^

**FIGURE 3:**
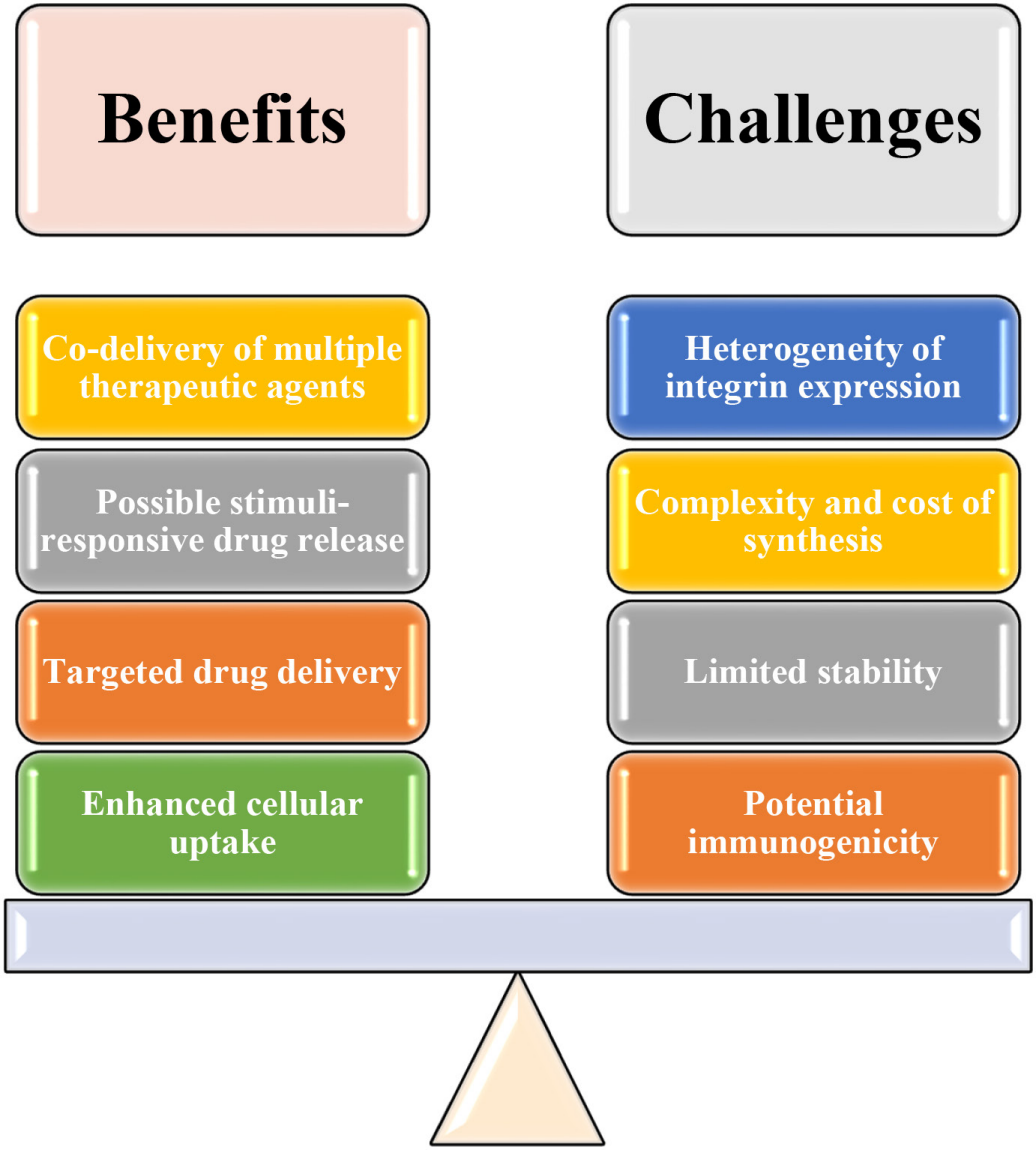
Possible benefits and challenges of RGD peptides.

RGD peptides are derived from natural proteins, which may trigger an immune response in some patients (Table 1, Figure 3). This could lead to the production of antibodies against the RGD peptide, potentially reducing the effectiveness of the therapy or causing adverse reactions.^77^, ^78^, ^79^, ^80^ Peptides, including RGD peptides, can be susceptible to degradation by proteases in the body. This may limit their stability and reduce their effectiveness in drug delivery.^81^, ^82^, ^83^ To overcome this issue, researchers often use modified or cyclic RGD peptides, which exhibit increased stability and resistance to protease degradation. The synthesis of RGD‐functionalized drug carriers can be complex and costly, particularly when multiple components, such as drugs, imaging agents, and stimuli‐responsive materials, are involved.^25^, ^84^, ^85^, ^86^ This may limit the widespread adoption of RGD peptide‐based drug delivery systems. Integrin expression can vary between different types of cancer and even between individual tumors of the same type.^66^, ^87^ This heterogeneity may affect the effectiveness of RGD peptide‐based drug delivery, as the therapy may be more effective in some patients than in others.

**TABLE 1:** The possible issues, effects, and possible related solutions.

RGD peptides		
Issues	Effect	Possible solutions
Induction of an immune response through T‐cell activation	Production of anti‐RGD antibodies	Using modified versions of the RGD peptide that cannot activate T‐cells or are less immunogenic.
	The immune response may affect the efficacy of RGD peptide‐based therapies	Pretreatment of patients with immunosuppressive drugs.
Repeated exposure to RGD peptide	Chronic immune responses and severe adverse reactions	Functionalizing RGD peptide with biocompatible polymers or coatings to reduce immune recognition and increase the lifespan of the peptide.
Altered conformation of the RGD peptide	The induction of new antigenic epitopes that can provoke an immune response	Choosing a suitable delivery system that can protect the RGD peptide from immune recognition.
Purity and quality of the RGD peptide	Presence of contaminating antigens that can trigger an immune response	Using high‐quality RGD peptide products that meet the regulatory standards for purity and absence of contaminants.
Short half‐life	Rapid degradation by proteases	Conjugation to protein carriers or nanoparticles to increase stability and half‐life.
Low solubility	Poor bioavailability	Chemical modifications to improve solubility and reduce protease susceptibility.
Poor stability in biological environments	Rapid degradation	Encapsulation in liposomes or other drug delivery systems to protect against degradation.
Lack of target specificity		Use of site‐specific targeting approaches.
High toxicity in some cases		Modification of the peptide sequence to reduce toxicity.
The complexity of the peptide structure and synthesis		Develop more efficient synthetic methods using specialized reagents and devices.
High synthesis cost and low yields.		Optimize the reaction parameters such as temperature, pH, and concentration to improve yields and reduce cost.
Difficulties in developing scalable synthesis methods.		Implement automation techniques to reduce handling errors and increase throughput.
Limited availability of amino acid building blocks.		Improve the availability and cost of amino acid building blocks by developing new synthetic routes or using alternative sources.
The need for multiple purification steps to obtain high purity.		Develop novel purification techniques such as immobilized‐metal affinity chromatography or reverse‐phase high‐performance liquid chromatography to reduce the number of steps required for purification.
Heterogeneity of integrin expression on different cell types and within a single cell type.		Using integrin‐specific antibodies or aptamers to capture or target specific integrin‐expressing cells.
Variable affinity and selectivity of RGD peptides for different integrin subtypes.		Design and optimize RGD peptides with higher affinity and selectivity for a specific integrin subtype by modifying the peptide structure.
RGD peptide internalization and degradation by cells.		Use modified RGD peptides or peptide conjugates that are resistant to degradation and can prolong integrin binding.
Competition for integrin binding by other ligands in the extracellular matrix.		Combine the RGD peptide with other proteins or peptides that can selectively compete for the binding sites of integrins and enhance RGD peptide binding.
Poor penetration of RGD peptides into solid tumors or tissues.		Combine the RGD peptide with nanocarriers, such as liposomes or nanoparticles, to improve delivery and penetration into target tissues.

## 4 POTENTIAL IMMUNOGENICITY

To overcome the potential immunogenicity of RGD peptides, altering the peptide structure can help reduce immunogenicity. For example, enlarging the peptide ring of c(RGDyK) by introducing an amino sequence serine‐glycine‐serine (SGS) has been shown to reduce the incidence of anaphylaxis after repeated intravenous c(RGDyKSGS)‐liposome stimulation.^79^ This modification was effective in reducing the incidence of anaphylaxis post the repeated intravenous c(RGDyKSGS)‐liposome stimulation. Therefore, the introduction of the SGS sequence into c(RGDyK)‐liposomes serves as a strategy to mitigate the immunogenicity‐associated issue and enhance the safety profile of these drug delivery systems. Incorporating RGD peptides into stealth lipids, such as polyethylene glycol (PEG)‐lipids, can help reduce immunogenicity by shielding the peptide from the immune system.^88^, ^89^, ^90^ This approach can also prolong the circulation time of the RGD peptide in the bloodstream, allowing for more effective delivery to the target site. A promising strategy for attenuating the immune response is presented when RGD peptide‐based lipids are combined with immunosuppressive agents. This approach was exemplified by the study mentioned in the provided document, where RGD‐modified lipid nanoparticles (LNPs), specifically RGD‐PEG‐lipid modified through the post‐insertion method, were utilized. This modification allowed the nanocarrier system to be preferentially targeted toward integrin‐expressing tumor endothelial cells (TECs). In a recent study, an immunosuppressive agent, aPD‐1 monoclonal antibody (mAb), was employed. The programmed cell death protein 1 (PD‐1) receptor, a key regulator of T‐cell responses, was inhibited by this antibody, thereby enhancing antitumor immune responses. By combining RGD‐modified LNPs with aPD‐1 mAb and siVegfr2 (small interfering RNA against Vegfr2), a reduction in tumor‐infiltrating lymphocytes (TILs), indicative of immune response mitigation, was achieved. Furthermore, vascular normalization was induced, and tumor growth was suppressed through this combination approach. It is worth noting that RGD‐PEG‐lipid was the lipid utilized in the study, and aPD‐1 mAb was the specific immunosuppressive agent employed. This strategy, which involves the utilization of RGD peptide‐based lipids and immunosuppressive agents, aligns with the broader goal of improving the efficacy of gene therapy while mitigating potential adverse immune reactions. It thus represents a promising avenue for further exploration in cancer treatment.^91^, ^92^ This approach can be particularly useful when the RGD peptide is used in combination with other therapeutic agents, such as small molecules or antibodies.^10^, ^93^, ^94^, ^95^ The choice of delivery system can have a significant impact on the immunogenicity of RGD peptides, which may include liposomes, nanoparticles, and micelles, etc. It is needed to find the most suitable option for reducing immunogenicity while maintaining therapeutic efficacy.^96^, ^97^, ^98^, ^99^ Considering the diversity of major histocompatibility complex (MHC) alleles in different populations and races, designing personalized peptides based on an individual's MHC profile can help reduce the risk of immunogenicity.^100^ It is essential to carefully evaluate each approach's benefits and drawbacks to determine the most suitable method for a specific application.

## 5 LIMITED STABILITY

The RGD peptide's poor solution stability can be overcome by cyclizing the linear RGD peptide.^101^, ^102^ It can improve its stability greatly by lowering conformational flexibility and making it less sensitive to proteolysis. A covalent link is formed between two amino acids in the linear sequence to accomplish cyclization. Multiple copies of RGD sequences incorporated into a single molecule can boost their affinity for integrin receptors, compensating for lower individual binding affinities. Multimeric structures incorporating surface‐bound RGD peptides, such as dendrimers or nanoparticles, have shown increased biological activity. The addition of polyethylene glycol (PEG) chains to the RGD peptide improves its solubility, decreases immunogenicity, and extends its circulation duration in vivo.^103^, ^104^, ^105^, ^106^ PEGylation protects peptides from enzymatic breakdown while retaining bioactivity. By embedding or conjugating RGD peptides onto biomaterial surfaces such as scaffolds, controlled release over time can be achieved which could enhance local concentration at local sites.^105^, ^106^, ^107^ Co‐administration or conjugation with serpins may protect the peptide against early hydrolysis by inhibiting the breakdown of enzymes.^108^, ^109^ Serpins, also known as serine protease inhibitors, are a superfamily of proteins that play a crucial role in regulating protease activity. They are characterized by a conserved structure and mechanism of action. Serpins inhibit serine proteases by forming a covalent complex with the protease, leading to its inactivation. This interaction involves a reactive center loop (RCL) within the serpin molecule that acts as a bait for the protease. Serpins are involved in various physiological processes, including blood clotting, immune response, and inflammation. They also have implications in diseases such as cancer, thrombosis, and neurodegenerative disorders.^109^ Chemical changes such as N‐methylation or D‐amino acid substitution to particular amino acids within an RGD motif may assist increase resistance to enzymatic cleavage without reducing receptor affinity. Non‐peptide analogs or peptidomimetics that replicate the structure and function of the RGD peptide can improve stability, bioavailability, and potency. When compared to endogenous peptides, these molecules are generally less vulnerable to proteolysis.^110^, ^111^, ^112^ It is feasible to overcome the restricted stability of RGD peptides while retaining their biological activity for diverse therapeutic purposes by using these tactics singly or in combination.

## 6 COMPLEXITY AND COST OF SYNTHESIS CHALLENGES OF RGD PEPTIDE

Due to the necessity for specialized modifications or multimeric structures, the synthesis of RGD peptides can be complicated, time‐consuming, and expensive. To address these obstacles, new RGD motifs with simpler structures or more accessible synthetic methods may provide equal biological activity while lowering synthesis complexity and expense.^4^, ^25^, ^113^, ^114^

Solid‐phase peptide synthesis (SPPS) provides for the effective step‐by‐step assembly of amino acids on a solid platform, eliminating purification stages and enhancing total yield. Automated SPPS synthesizers reduce manual effort and human error even further. Adjusting reaction parameters such as temperature, solvent choice, coupling reagents, or protecting groups may increase peptide synthesis efficiency and prevent side reactions that cause contaminants. Continuous improvement in production procedures through optimization studies will increase process efficiency, resulting in less waste, higher product quality, and lower cost per unit. Scaling up production processes by investing in larger reactors/fermenters will result in lower cost per unit product as fixed expenses are dispersed over a larger number of units produced.^115^, ^116^, ^117^, ^118^

For longer or more complex RGD‐containing peptides or proteins, chemical ligation techniques like native chemical ligation (NCL) enable the convergent assembly of multiple smaller peptide fragments with greater synthetic accessibility.^119^, ^120^, ^121^, ^122^ Producing RGD peptides using recombinant DNA technology in bacterial (e.g., E.coli), yeast (e.g., Pichia pastoris), insect cells (e.g., baculovirus system), or mammalian cell cultures can provide higher yields compared to traditional chemical synthesis methods at lower costs per batch.^123^, ^124^, ^125^, ^126^, ^127^ Efficient bioconjugation strategies such as click chemistry allow for site‐specific attachment of functional moieties like PEG chains without requiring additional protection/deprotection steps during peptide synthesis.^38^, ^128^, ^129^, ^130^

By adopting these strategies individually or in combination, it is possible to overcome the complexity and cost challenges associated with the synthesis of RGD peptides without compromising their therapeutic potential.

## 7 HETEROGENEITY OF INTEGRIN EXPRESSION CHALLENGE OF RGD PEPTIDE

Heterogeneous expression of integrins on different cell types and tissues can impact the targeting specificity and therapeutic efficacy of RGD peptides. To overcome this challenge, design RGD peptide derivatives that selectively target specific integrin subtypes overexpressed in pathological conditions such as cancer or inflammation.^131^ This can be achieved by modifying amino acid sequences, incorporating additional functional groups, or using peptidomimetics.

The application of targeted extracellular matrix (ECM)‐derived peptides for promoting neovascularization in a rodent model of myocardial infarction is the focus of the document. One specific approach mentioned in the document is the conjugation of targeting moieties, such as antibodies or aptamers, to RGD peptide constructs. The interaction of the RGD peptide sequence with integrin receptors on the surface of cells is well‐known. By conjugating targeting moieties, such as antibodies or aptamers, to RGD peptide constructs, the specificity of the peptides toward cells with co‐expression patterns related to disease conditions is increased. This results in an increased likelihood of the peptides binding to and interacting with cells specifically involved in the disease process, such as cells in the infarcted area of the heart. The reduction of off‐target effects on normal cells is achieved by increasing the specificity of the peptides. This is important as it minimizes any potential negative impact on healthy cells and tissues. The conjugation of targeting moieties to RGD peptide constructs enables a more precise and targeted delivery of the peptides to the desired cells, thereby enhancing their therapeutic potential. Hence, a promising strategy for improving the specificity and effectiveness of ECM‐derived peptides in promoting neovascularization in the context of myocardial infarction is offered by the approach of conjugating targeting moieties to RGD peptide constructs. Conjugating other targeting moieties (e.g., antibodies, aptamers) to RGD peptide construct increases specificity toward cells with co‐expression patterns related to disease conditions while reducing off‐target effects on normal cells.^132^ Developing stimuli‐responsive drug delivery systems is also helpful because they can release their cargo only under certain physiological/pathological conditions like acidic tumor microenvironments or enzymatic cleavage by proteases specifically upregulated in diseased tissue.^31^, ^133^, ^134^, ^135^ Nanoparticles and nanocarriers could utilize engineered surface‐bound RGD motifs for enhanced cellular uptake via receptor‐mediated endocytosis while carrying a therapeutic payload within the core/shell matrix.^134^, ^136^ Designing prodrugs that are activated upon binding to a specific integrin subtype through enzymatic cleavage or conformational changes, etc. resulting from receptor‐ligand interaction can release an active RGD molecule at targeted sites.^137^, ^138^, ^139^ A combination of RGD peptide‐based therapies with other treatments like chemotherapy agents or immune checkpoint inhibitors could enhance treatment efficacy while potentially mitigating resistance mechanisms associated with single‐agent therapy.^26^, ^140^, ^141^, ^142^

Identifying biomarkers indicative of high integrin expression levels corresponding to likely responders allows for better patient stratification and personalized treatment approaches. Determining the optimal dosing, frequency, and routes of administration would maximize therapeutic efficacy while minimizing off‐target effects on normal tissues with lower integrin expression levels.^142^, ^143^, ^144^, ^145^ Utilizing imaging techniques like PET or MRI in conjunction with RGD‐based probes for visualizing changes in integrin expression during therapy allows early identification of responders and facilitates timely adjustments to the treatment plan if necessary.^53^, ^146^, ^147^, ^148^ By employing these strategies individually or in combination, it is possible to overcome challenges posed by heterogeneous integrin expression when using RGD peptides for targeted therapies while maximizing their clinical potential.

## 8 SOME AVAILABLE RGD‐BASED DRUGS

RGD peptides are short peptide fragments derived from the amino acid sequence of several extracellular matrix proteins, such as fibrinogen, fibronectin, vitronectin, collagen, and laminin. They are widely present in fibronectin, laminin, fibrinogen, osteopontin, and vitronectin.

RGD can be divided into two types:

1. RGD: This is a tripeptide sequence of RGD.
2. RGD polypeptide: This is a functional peptide containing RGD.

Depending on the application and the integrin targeted, RGD can be chemically modified or replaced by a similar peptide which promotes cell adhesion. For example, RGD peptides can be cyclized, or made into a cyclic compound, via disulfide, thioether, or rigid aromatic ring linkers. This leads to an increase in binding affinity and selectivity for integrin αVβ3 relative to αIIBβ3.

Another type of RGD‐derived peptide is the “internalizing RGD” or “iRGD.” This peptide has the ability to recognize the integrin receptor on the cancer cell surface like its ancestor with an additional outstanding feature to penetrate to extravascular space of tumor and ability to penetrate to cancer cells unlike the original peptide.^149^, ^150^, ^151^, ^152^, ^153^, ^154^

**FIGURE 4:**
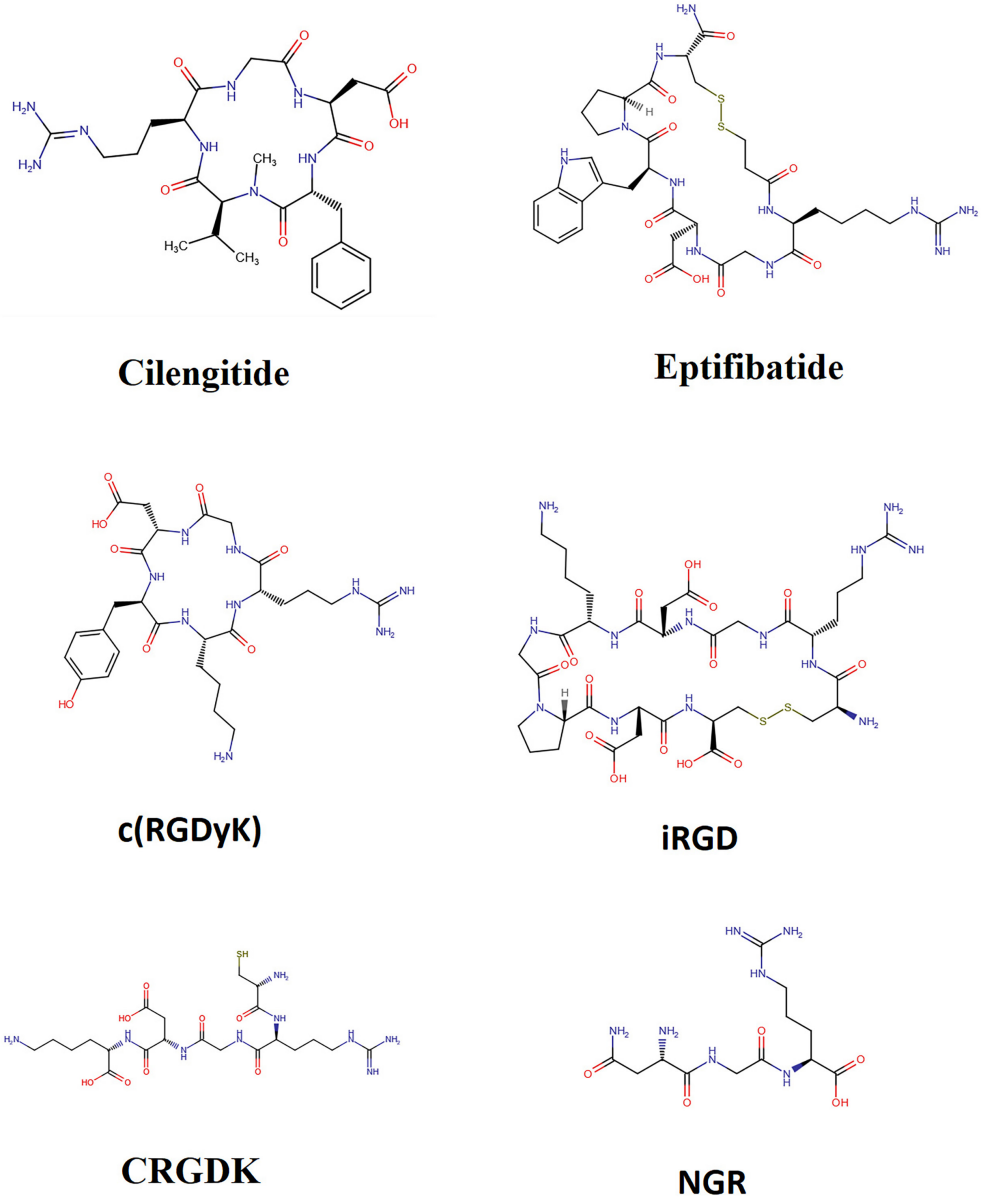
Stucture of some RGD‐peptides.

Cilengitide is a cyclic RGD peptide that targets αvβ3 and αvβ5 integrins (Figure 4). It has been investigated as a potential treatment for glioblastoma, a type of brain cancer. Cilengitide has shown promising results in preclinical studies (Table 2), but its efficacy in clinical trials has been limited. One disadvantage of cilengitide is its potential immunogenicity, which may reduce its effectiveness in some patients.^71^, ^155^, ^156^, ^157^ Eptifibatide is also a cyclic RGD peptide that targets αIIbβ3 integrin, which is involved in platelet aggregation. Eptifibatide is used as an antiplatelet agent to prevent blood clots in patients with acute coronary syndrome or undergoing percutaneous coronary intervention. One advantage of eptifibatide is its rapid onset of action, which can help prevent thrombotic events.^158^, ^159^, ^160^, ^161^ Abegrin is a cyclic RGD peptide that α_v_β3 integrin, which is overexpressed in several types of cancer. Abegrin has been investigated as a potential treatment for various cancers, including breast cancer and pancreatic cancer. The associated clinical trials can be found in Table 3. One advantage of Abegrin is its high specificity for α_v_β3 integrin, which can improve the efficacy of the therapy.^9^, ^162^, ^163^, ^164^

**TABLE 2:** Cilengitide in preclinical studies.

NCT number	Study title	Study status	Brief summary	Study Results	Phases	Start Date	Completion Date
NCT00842712	Cilengitide and Cetuximab in Combination With Platinum‐based Chemotherapy as First‐line Treatment for Subjects With Advanced Non Small Cell Lung Cancer (NSCLC)	Completed	Primary objective of the study's safety run‐in: ‐ To determine the maximum tolerated dose (MTD) of cilengitide in combination with cetuximab, and platinum‐based chemotherapy (cisplatin/vinorelbine or cisplatin/gemcitabine). Primary objective of the study's randomization part: ‐ To assess the efficacy of cilengitide in combination with cetuximab and platinum‐based chemotherapy (cisplatin/vinorelbine or cisplatin/gemcitabine) compared to cetuximab and platinum‐based chemotherapy alone in terms of progression‐free survival (PFS) time. Study design and plan: This is a multicenter, open‐label, randomized, and controlled phase II study with a safety run‐in part in subjects with advanced non‐small cell lung cancer (NSCLC). During the safety run‐in, the regimen was intensified stepwise by cohort (cilengitide intravenous \[iv\] 1000 milligram \[mg\] to 2000 mg twice a week) in a classical 3 + 3 subjects (for each platinum‐based chemotherapy regimens separately) approach with predefined dose‐ and schedule reduction rules. In the safety run‐in 12 subjects were included and evaluated for safety and feasibility of different escalating doses of cilengitide administered twice weekly in combination with cetuximab, cisplatin and vinorelbine or gemcitabine. After completion of the safety run‐in, the randomized part will be started, during which all subjects will receive cetuximab and platinum‐based chemotherapy (cisplatin/vinorelbine or cisplatin/gemcitabine). Subjects will be centrally randomized on a 1:1 basis to either Group A or C; Group B will be closed with implementation of Amendment No. 4 (dated 20 December 2010): â€¢ Group A: Cilengitide 2000 mg once weekly (Days 1, 8, and 15 of every 3‐week chemotherapy cycle) in combination with cetuximab and platinum‐based chemotherapy that will consist of the following: * Cetuximab once weekly (Days 1, 8, and 15), plus cisplatin on Day 1 and vinorelbine on Days 1 and 8 of every 3‐week chemotherapy cycle, or * Cetuximab once weekly (Days 1, 8, and 15), plus cisplatin on Day 1 and gemcitabine on Days 1 and 8 of every 3‐week chemotherapy cycle. The decision which of the 2 chemotherapy regimens will be applied for a given subject is at the discretion of the treating investigator. â€¢ Group B: Cilengitide 2000 mg twice weekly (Days 1, 4, 8, 11, 15, and 18 of every 3‐week chemotherapy cycle) in combination with cetuximab and platinum‐based chemotherapy as described for Group A. Group B will be closed with implementation of Amendment No. 4 (global, dated December 20, 2010). Subjects randomized to Group B before implementation of Amendment No 4 will continue to be treated as planned. â€¢ Group C: cetuximab and platinum‐based chemotherapy as described for Group A Chemotherapy will be given until radiographically documented progressive disease (PD) or unacceptable toxicity but for no more than 6 cycles. Cilengitide and cetuximab will be given until radiographically documented PD or unacceptable toxicity. Randomization will be performed centrally using an interactive voice/web response system (IXRS). A stratified block randomization procedure will be employed using chosen first‐line chemotherapy (cisplatin/vinorelbine vs. cisplatin/gemcitabine) as stratification criterion.	YES	PHASE2	2009–02	2013–07
NCT00077155	Cilengitide (EMD 121974) in Treating Patients With Advanced Solid Tumors or Lymphoma	Completed	This Phase I trial is studying the side effects and best dose of EMD 121974 in treating patients with solid tumors or lymphoma. Cilengitide (EMD 121974) may stop the growth of cancer cells by stopping blood flow to the cancer.	NO	PHASE1	2003–12	
NCT00112866	Cilengitide in Treating Patients Who Are Undergoing Surgery for Recurrent or Progressive Glioblastoma Multiforme	Terminated	Cilengitide may stop the growth of glioblastoma multiforme by blocking blood flow to the tumor. Giving cilengitide before and after surgery may be an effective treatment for glioblastoma multiforme. This Phase II trial is studying how well cilengitide works in treating patients who are undergoing surgery for recurrent or progressive glioblastoma multiforme.	YES	PHASE2	2005–01	2009–03
NCT01122888	Cilengitide and Sunitinib Malate in Treating Patients With Advanced Solid Tumors or Glioblastoma Multiforme	Terminated	This clinical trial is studying how well giving cilengitide together with sunitinib malate works in treating patients with advanced solid tumors or glioblastoma multiforme. Cilengitide and sunitinib malate may stop the growth of tumor cells by blocking blood flow to the tumor. Giving cilengitide together with sunitinib malate may kill more tumor cells. Studying samples of blood in the laboratory from patients receiving cilengitide and sunitinib malate may help doctors understand the effect of these drugs on biomarkers.	NO	PHASE1	2009–12	2015–04
NCT00085254	Cilengitide, Temozolomide, and Radiation Therapy in Treating Patients With Newly Diagnosed Glioblastoma Multiforme	Completed	Cilengitide may stop the growth of cancer by stopping blood flow to the tumor. Drugs used in chemotherapy, such as temozolomide, work in different ways to stop the growth of tumor cells, either by killing the cells or by stopping them from dividing. Radiation therapy uses high‐energy x‐rays to damage tumor cells. Giving cilengitide together with temozolomide and radiation therapy may kill more tumor cells. This randomized phase I/II trial is studying the side effects and best dose of cilengitide when given together with temozolomide and radiation therapy and to compare how well they work in treating patients with newly diagnosed glioblastoma multiforme.	YES	PHASE1|PHASE2	2005–04	2012–11
NCT00082875	Cilengitide in Treating Patients With Unresectable or Metastatic Melanoma	Terminated	This randomized phase II trial is studying how well cilengitide works in treating patients with unresectable Stage III or Stage IV melanoma. Cilengitide may stop the growth of melanoma by stopping blood flow to the tumor.	NO	PHASE2	2004–03	
NCT00813943	Cilengitide, Temozolomide, and Radiation Therapy in Treating Patients With Newly Diagnosed Glioblastoma and Unmethylated Gene Promoter Status	Completed	CORE is a Phase 2 clinical trial in newly diagnosed glioblastoma in subjects with an unmethylated O6‐methylguanine‐deoxyribonucleic acid methyltransferase (MGMT) gene promoter in the tumor tissue. The MGMT gene promoter is a section of deoxyribonucleic acid (DNA) that acts as a controlling element in the expression of MGMT. Methylation of the MGMT gene promoter has been found to appear to be a predictive marker for benefit from temozolomide (TMZ) treatment. In a safety run‐in period in dedicated study centers, the safety and tolerability of cilengitide given as an intense treatment in combination with the first part of standard therapy will be assessed. Thereafter the trial will investigate the overall survival and progression‐free survival in subjects receiving two different regimens of cilengitide in combination with standard treatment versus standard treatment alone.	YES	PHASE2	2009–03	2013–08
NCT00103337	Cilengitide in Treating Patients With Metastatic Prostate Cancer	Completed	This randomized phase II trial is studying how well cilengitide works in treating patients with metastatic prostate cancer. Cilengitide may stop the growth of prostate cancer by blocking blood flow to the tumor.	NO	PHASE2	2005–01	
NCT00006222	EMD 121974 in Treating Patients With HIV‐Related Kaposi's Sarcoma	Terminated	Phase I trial to study the effectiveness of EMD 121974 in treating patients who have HIV‐related Kaposi's sarcoma. EMD 121974 may stop the growth of Kaposi's sarcoma by stopping blood flow to the tumor.	NO	PHASE1	2000–09	2001–03
NCT01558687	Cilengitide Imaging Trial in Glioblastoma	Terminated	The main purpose of this clinical trial is to find out if cilengitide has an effect on brain tumor cells but also particularly on the blood vessels supplying the tumor with nutrient and oxygen in patients newly diagnosed with non‐resectable (inoperable) glioblastoma. In addition, this clinical trial will investigate if the addition of cilengitide in combination with standard treatment prolongs life in patients with non‐resectable glioblastoma. Similarly, the duration of response of the cancer to this treatment and the side effects of the therapy will be analyzed. Furthermore, additional data on how the body deals with this substance will be collected (this is called pharmacokinetics or pharmacokinetic (PK) analysis). In this clinical trial the investigators would also like to learn more about the disease and the response to the experimental medication by measuring certain “markers.” This imaging trial will investigate the biological effects of cilengitide monotherapy on the tumor microvascular function and tumor viability in a homogenous non‐pretreated subject population with newly diagnosed gliobastoma (GBM). The purpose of this clinical trial is to study the effect that cilengitide may have on certain markers of cancer in your tumor and/or blood and to learn if there are any disease‐related markers that could help in predicting how subjects respond to the administration of cilengitide. The investigators anticipate that approximately 30 subjects will participate in this clinical trial. The clinical trial will be conducted in approximately 4 medical centers in the following countries: Germany, Poland, and Switzerland. The investigators anticipate the clinical trial will last until the end of 2013. Your participation in the trial may last up to 86 weeks.	NO	PHASE1	2012–08	2013–02
NCT01165333	Cilengitide in Combination With Irradiation in Children With Diffuse Intrinsic Pontine Glioma	Completed	The aim of the study is to determine the safety of cilengitide in combination with radiation therapy.	NO	PHASE1	2010–08	2015–03
NCT00705016	Cilengitide in Recurrent and/or Metastatic Squamous Cell Carcinoma of the Head and Neck (SCCHN)	Completed	The purpose of this open‐label, randomized, controlled, Phase 1/2 study of the integrin inhibitor cilengitide is to evaluate the safety and efficacy of the combination of different regimens of cilengitide added to cisplatin, 5‐fluorouracil (5‐FU), and cetuximab in participants with recurrent/metastatic squamous cell carcinoma of the head and neck (SCCHN). The Phase 1 part was conducted in dedicated study centers. In the Phase 2 part of this trial, cilengitide is administered at two different doses to two experimental groups. The third group will only receive cisplatin, 5‐FU and cetuximab. In the Phase 1 part of this trial, the dose of cilengitide in combination with cisplatin, 5‐FU, and cetuximab was determined. Cilengitide is an experimental anticancer substance interacting with so‐called integrins. Integrins are protein molecules that are known to be present on the surface of certain cancer cells. Integrins are also found on certain cells that belong to growing blood vessels (endothelial cells). Integrins potentially facilitate the blood vessels' support of the tumor (angiogenesis) as well as the tumor's growth and further spread throughout the body (metastasis). By inhibiting integrins on the tumor cell surface, cilengitide potentially kills cancer cells, and potentially sensitizes cancer cells to other co‐administered therapeutics. By inhibiting integrins on the endothelial cell surface, it potentially inhibits the ingrowth of additional blood vessels toward the tumor. Cilengitide is given as an intravenous infusion (given by a drip in one vein of your arm). If any unacceptable side effect occurs, treatment with the study drug will be stopped.	YES	PHASE1|PHASE2	2008–10	2013–06
NCT01044225	Effect of Radiation Therapy Plus Temozolomide Combined With Cilengitide or Cetuximab on the 1‐year Overall Survival of Patients With Newly Diagnosed MGMT‐promoter Unmethylated Glioblastoma	Terminated	The investigators propose to conduct a multicenter, open‐label, randomized, phase II study in patients with newly diagnosed glioblastoma (CeCil). Patients should meet all eligibility criteria for the CENTRIC phase III trial at the exception that no MGMT‐promoter methylation could be demonstrated. The treatment backbone in both study arms will consist of postoperative radiation therapy with concomitant daily temozolomide, followed by 6 cycles of temozolomide according to a 21 out of 28 days regimen (as in the experimental arm of the RTOG 0525/EORTC 26052–22,053 phase III study). In study arm (A) cilengitide (at a dose of 2000 mg by iv administration, 2x/week) will be added to this backbone while in the second study arm (B), cetuximab will be added (at an initial dose of 400 mg/mÂ^2^ administered by intravenous infusion over 2 hours and followed by a weekly dose of 250 mg/mÂ^2^ iv over 1 h). In both study arms, treatment will be administered for 52 consecutive treatment weeks. The 1 year overall survival (1y‐OS) following randomization will serve as the primary endpoint in both study arms.	NO	PHASE2	2009–09	2011–09
NCT00689221	Cilengitide, Temozolomide, and Radiation Therapy in Treating Patients With Newly Diagnosed Glioblastoma and Methylated Gene Promoter Status	Completed	CENTRIC is a Phase 3 clinical trial assessing efficacy and safety of the investigational integrin inhibitor, cilengitide, in combination with standard treatment versus standard treatment alone in newly diagnosed glioblastoma subjects with a methylated O6‐methylguanine‐deoxyribonucleic acid methyltransferase (MGMT) gene promoter in the tumor tissue. The MGMT gene promoter is a section of deoxyribonucleic acid (DNA) that acts as a controlling element in the expression of MGMT. Methylation of the MGMT gene promoter has been found to be a predictive marker for benefit from temozolomide (TMZ) treatment.	YES	PHASE3	2008–09	2013–08
NCT00121238	Cilengitide in Treating Patients With Prostate Cancer	Completed	This phase II trial is studying how well cilengitide works in treating patients with prostate cancer. Cilengitide may stop the growth of prostate cancer by blocking blood flow to the tumor	YES	PHASE2	2005–01	2015–11
NCT00089388	Cilengitide in Treating Patients With Acute Myeloid Leukemia	Terminated	This randomized phase II trial is studying how well cilengitide works in treating patients with acute myeloid leukemia. Cilengitide may stop the growth of cancer cells by blocking the enzymes necessary for their growth	NO	PHASE2	2004–07	
NCT00979862	Cediranib Maleate and Cilengitide in Treating Patients With Progressive or Recurrent Glioblastoma	Completed	This Phase I trial is studying the side effects and best dose of cediranib maleate when given together with cilengitide in treating patients with progressive or recurrent glioblastoma. Cediranib maleate and cilengitide may stop the growth of tumor cells by blocking blood flow to the tumor. Giving cediranib maleate together with cilengitide may kill more tumor cells.	NO	PHASE1	2010–03	2014–02
NCT00679354	Cilengitide in Treating Younger Patients With Recurrent or Progressive High‐Grade Glioma That Has Not Responded to Standard Therapy	Completed	This Phase II trial studies how well cilengitide works in treating younger patients with recurrent or progressive high‐grade glioma that has not responded to standard therapy. Cilengitide may stop the growth of tumor cells by blocking blood flow to the tumor.	YES	PHASE2	2008–06	2011–07
NCT00006093	EMD 121974 in Treating Patients With Progressive or Recurrent Glioma	Completed	RATIONALE: EMD 121974 may stop the growth of cancer by stopping blood flow to the tumor. PURPOSE: Phase I/II trial to study the effectiveness of EMD 121974 in treating patients who have progressive or recurrent malignant glioma.	NO	PHASE1|PHASE2	2000–09	2006–10
NCT00063973	Cilengitide in Treating Children With Refractory Primary Brain Tumors	Completed	This phase I trial is studying the side effects and best dose of cilengitide in treating children with recurrent, progressive, or refractory primary CNS tumors. Cilengitide may slow the growth of brain cancer cells by stopping blood flow to the tumor.	NO	PHASE1	2003–07	
NCT00022113	EMD 121974 in Treating Patients With Advanced Solid Tumors	Completed	Phase I trial to study the effectiveness of EMD 121974 in treating patients who have advanced solid tumors. EMD 121974 may slow the growth of solid tumors by stopping blood flow to the tumor	NO	PHASE1	2001–05	
NCT01276496	Weekly Doses of Cilengitide and Paclitaxel in Treating Patients With Advanced Solid Tumors That Cannot Be Removed by Surgery	Completed	This Phase I trial studies the side effects and the best dose of cilengitide when given together with paclitaxel weekly in treating patients with solid tumors that have spread nearby or to other areas of the body and cannot be removed by surgery. Cilengitide may stop the growth of tumor cells by blocking blood flow to the tumor. Drugs used in chemotherapy, such as paclitaxel, work in different ways to the stop the growth of tumor cells, either by killing the cells or by stopping them from dividing. Giving cilengitide together with paclitaxel may kill more tumor cells.	NO	PHASE1	2010–12	
NCT00093964	Cilengitide (EMD 121974) for Recurrent Glioblastoma Multiforme (Brain Tumor)	Completed	This study will investigate clinical activity, safety, and tolerability of the antiangiogenic compound cilengitide (EMD 121974) in the treatment of first recurrence of glioblastoma multiforme (GBM).	YES	PHASE2	########	########
NCT01118676	Cilengitide Together With Radiochemotherapy in Patients With Locally Advanced Non Small Cell Lung Cancer	Completed	This is a two‐center study which includes 24 patients maximum on 36 months: 24 months accrual ‐ 12 months follow‐up. Eligible patients are included according to a standard 3 + 3 design. Patients included in the trial will be treated with a combination of radiochemotherapy (standard radiotherapy of 66 Gy, 2 Gy per daily fraction, and cisplatin and vinorelbine‐based chemotherapy). Cilengitide will be administered alone as continuous infusion two weeks before the radiochemotherapy and will then be continued during radiochemotherapy as continuous infusion. The dose levels investigated will be applied to the continuous administration (a maximum of 4 dose levels). After the end of concomitant radiochemotherapy, cilengitide will be administered i.v. at a dose of 2000 mg twice weekly until the end of chemotherapy. The dose of Cilengitide administered after radiotherapy will not be increased. 4 dose levels are defined:12, 18, 27 et 40 mg/hour.	NO	PHASE1	2010–03	2015–04
NCT00884598	Cilengitide and Whole‐Brain Radiation Therapy in Treating Patients With Brain Metastases From Lung Cancer	Unknown	RATIONALE: Cilengitide may stop the growth of brain metastases by blocking blood flow to the tumor. Radiation therapy uses high energy x‐rays to kill tumor cells. Giving cilengitide together with radiation therapy may kill more tumor cells. PURPOSE: This Phase I trial is studying the side effects and best dose of cilengitide when given together with whole‐brain radiation therapy in treating patients with brain metastases from lung cancer.	NO	PHASE1	2008–12	2011–12
NCT01124240	Temozolomide and Procarbazine With Cilengitide for Patients With Glioblastoma Multiforme Without Methylation of the MGMT Promoter Gene	Unknown	Cilengitide 2000 mg flat iv twice weekly is administered over a period of 18 months without interruption. Starting one week after the initiation of Cilengitide, RTX (60 Gy, 2 Gy per fraction) with concurrent daily temozolomide (60 mg/m2 p.o.) and daily procarbazine (PCB, 50 mg p.o. if BSA\ < 1.7; 100 mg p.o. if BSA 1.7) is given over a period of 6 weeks (RTX Monday to Friday, both TMZ and PCB 7 days a week). After a break of 4 weeks, adjuvant TMZ (50 mg/m2 p.o in first cycle, 60 mg/m2 p.o. in subsequent cycles) and PCB (50 mg p.o. if BSA \ < 1.7; 100 mg p.o. if BSA 1.7) are then given daily D1 to 20. This TMZ/PCB cycle is repeated every 28 days over a total period of 6 cycles.	NO	PHASE2	2009–11	2014–01
NCT00004258	EMD 121974 in Treating Patients With Locally Advanced or Metastatic Cancer	Completed	RATIONALE: EMD 121974 may stop the growth of cancer by stopping blood flow to the tumor. PURPOSE: Phase I trial to study the effectiveness of EMD 121974 in treating patients who have locally advanced or metastatic cancer.	NO	PHASE1	1999–12	2001–09
NCT01782976	Ph II Cilengitide Plus Bevacizumab for Recurrent Glioblastoma (GBM)	Withdrawn	The goal of this clinical research study is to learn if cilengitide given in combination with bevacizumab can help to control glioblastoma. The safety of this drug combination will also be studied. Cilengitide is designed to block the flow of blood to cancer cells, which may help to slow or block the growth of cancer. Bevacizumab is designed to block the growth of new blood vessels, which may help to slow or block the growth of cancer.	NO	PHASE2	2013–06	
NCT01517776	Cilengitide and Metronomic Temozolomide for Relapsed or Refractory High Grade Gliomas or Diffuse Intrinsic Pontine Gliomas in Children and Adolescents	Terminated	The primary objective of this study is to evaluate the efficacy of a combined treatment with cilengitide and metronomic oral temozolomide as measured by 6 months overall survival (OS) after diagnosis of relapse or tumor progression in children and adolescents with relapsed or refractory high‐grade malignant glioma and diffuse intrinsic pontine glioma. Secondary objectives include: 1. To evaluate the safety and toxicity of the study treatment by common toxicity criteria (CTC; version 4.0). 2. To assess * the response rates at 6 months (continuous complete response = CCR, complete response = CR, partial response = PR, stable disease = SD, progressive disease = PD) and * progression‐free survival (PFS) at 6 months, and * response rates, OS, and PFS at 12 months after relapse diagnosis or diagnosis of tumor progression. Response will be presented including histopathological variants. 3. To assess the pharmacokinetics of cilengitide administered as part of the study treatment. Indication and study population for this trial: Treatment of relapsed or refractory high grade gliomas and diffuse intrinsic pontine gliomas in pediatric patients 3 years and\<18 years of age. Patients included in the study receive * Cilengitide 1800 mg/mÂ^2^ iv twice weekly * Temozolomide 75 mg/mÂ^2^/d p.o. for 6 weeks, followed by 1 week rest with a mandatory platelet‐count dependent dose adaptation rule: mandatory blood counts twice weekly: Platelets stop temozolomide until platelet recovery * Study treatment in the individual patient is scheduled for 1 year unless tumor progression or excessive toxicity occurs. However, study treatment may be extended beyond 1 year upon individual decision.	NO	PHASE2	2012–01	2014–04

**TABLE 3:** Abegrin in clinical trials.

NCT number	Study title	Study status	Brief summary	Study Results	Phases	Start Date	Completion Date
NCT00049712	Monoclonal Antibody Therapy in Treating Patients With Refractory Advanced Solid Tumors or Lymphoma	Completed	RATIONALE: Monoclonal antibodies can locate cancer cells and either kill them or deliver cancer‐killing substances to them without harming normal cells. PURPOSE: Phase I trial to study the effectiveness of monoclonal antibody therapy in treating patients who have refractory advanced solid tumors or lymphoma.	NO	PHASE1	2002–10	2006–02
NCT00263783	Phase I Trial of Weekly MEDI‐522 in Patients With Refractory Solid Tumors	Completed	To determine the safety profile of single and multiple doses of MEDI522 in patients with refractory solid tumors.	NO	PHASE1	2001–03	2002–06
NCT00284817	Phase I Study of MEDI522 in Patients With Irinotecan‐Refractory Advanced Colorectal Cancer	Completed	#NAME?	NO	PHASE1|PHASE2	2001–07	2005–05
NCT00684996	Bevacizumab With or Without MEDI‐522 in Treating Patients With Unresectable or Metastatic Kidney Cancer	Terminated	This Phase I/randomized Phase II trial is studying the side effects and best dose of bevacizumab and to see how well it works when given together with or without MEDI‐522 in treating patients with unresectable or metastatic kidney cancer. Monoclonal antibodies, such as bevacizumab and MEDI‐522, can block tumor growth in different ways. Some block the ability of tumor cells to grow and spread. Others find tumor cells and help kill them or carry tumor‐killing substances to them. Bevacizumab and MEDI‐522 may also stop the growth of tumor cells by blocking blood flow to the tumor. It is not yet known whether bevacizumab is more effective when given together with or without MEDI‐522 in treating kidney cancer.	YES	PHASE1|PHASE2	2008–06	2010–10
NCT00111696	Study of the Tumor Saturation and Biological Activity of MEDI‐522 (Abergrin) in Patients With Advanced Malignant Melanoma	Completed	To describe the tumor tissue saturation by MEDI‐522 in patients with advanced malignant melanoma.	NO	PHASE1	2005–05	2007–11
NCT00066196	Evaluating The Antitumor Activity Of MEDI‐522 With Or Without Dacarbazine In Patients With Metastatic Melanoma	Completed	The primary objectives of this study are: * To explore the antitumor activity of MEDI‐522 Â ± DTIC in patients with metastatic melanoma. * To determine the safety of MEDI‐522 Â ± DTIC in this patient population.	NO	PHASE2	2003–08	2007–06
NCT00027729	Monoclonal Antibody Therapy in Treating Patients With Advanced Colorectal Cancer	Completed	RATIONALE: Monoclonal antibodies can locate tumor cells and either kill them or deliver tumor‐killing substances to them without harming normal cells. PURPOSE: Phase I/II trial to study the effectiveness of monoclonal antibody therapy in treating patients who have advanced colorectal cancer that has not responded to irinotecan.	NO	PHASE1|PHASE2	2001–06	2004–11
NCT00072930	MEDI‐522 in the Treatment of Patients With Metastatic Androgen‐Independent Prostate Cancer	Completed	The primary objectives of this study are: 1. To explore the antitumor activity of MEDI‐522 in combination with docetaxel, prednisone, and zoledronic acid in patients with metastatic Androgen‐Independent Prostate Cancer (AIPC); and 2. To summarize the safety of MEDI‐522 in combination with docetaxel, prednisone, and zoledronic acid in this patient population.	NO	PHASE2	2003–12	2007–06

## 9 RGD‐BASED DRUGS IN CLINICAL TRIALS

The clinical studies focus on various types of cancer, including ovarian cancer, lung cancer, head and neck cancer, glioblastoma, non‐small cell lung cancer, cervical cancer, and others. Each study aims to evaluate the specific application of the drugs in the context of these cancer types. The studies utilize different imaging techniques, such as PET/CT (positron emission tomography/computed tomography) and PET/MRI (positron emission tomography/magnetic resonance imaging), to assess tumor characteristics, angiogenesis, and response to treatment. These imaging techniques provide valuable information about the distribution and activity of the drugs within the body. Some studies have secondary objectives, such as evaluating immunologic responses, determining potential toxicities, assessing the predictive value of imaging techniques, and correlating imaging parameters with clinical treatment response (Table 4).

**TABLE 4:** RGD peptides in clinical trials.

NCT number	Study title	Study status	Brief summary	Study Results	Phases	Start Date	Completion Date
NCT03393689	RGD PET/MRI in Sporadic Vestibular Schwannoma	Unknown	The aim of this non‐randomized, prospective study is to investigate the applicability and prognostic value of angiogenesis PET/MR with the radioligand 68Ga‐NODAGA‐ E\[c(RGDyK)\]2 in patients with sporadic vestibular schwannomas.	NO	PHASE2	1/2/2018	1/2/2021
NCT00562003	Safety Study of a Genetically Modified Adenovirus in Ovarian Cancer Patients	Completed	The primary purpose of this study is to determine the maximally tolerated dose and spectrum of toxicities encountered with intraperitoneal delivery of a RGD modified conditionally replicative adenovirus (Ad5‐Delta 24RGD) in patients with recurrent ovarian cancer. Secondary objectives: * To determine the biologic effects encountered with intraperitoneal delivery of Ad5‐Delta 24RGD in patients with recurrent ovarian cancer cells. * To determine immunologic response generated against Ad5‐Delta 24RGD when administered intraperitoneally to patients with recurrent ovarian adenocarcinoma. * To determine potential clinical activity of Ad5‐Delta 24RGD when administered intraperitoneally to patients with recurrent ovarian adenocarcinoma.	NO	PHASE1	2007–06	2010–06
NCT01806675	18F‐FPPRGD2 PET/CT or PET/MRI in Predicting Early Response in Patients With Cancer Receiving Anti‐Angiogenesis Therapy	Completed	The purpose of the study is to conduct research of a new PET radiopharmaceutical in cancer patients. The uptake of the novel radiopharmaceutical 18F‐FPPRGD2 will be assessed in study participants with glioblastoma multiforme (GBM), gynecological cancers, and renal cell carcinoma (RCC) who are receiving antiangiogenesis treatment.	YES	PHASE1|PHASE2	3/4/2013	2019–04
NCT05515783	68Ga‐FAP‐RGD PET/CT: Dosimetry and Preliminary Clinical Translational Studies	Recruiting	As an new dual targeting PET radiotracer, 68Ga‐FAP‐RGD is promising as an excellent imaging agent applicable to various cancers. In this study, we observed the safety, biodistribution and radiation dosimetry of 68Ga‐FAP‐RGD in patients with various types of cancer and compared them with the results of 68Ga‐FAPI‐02 or 18F‐FDG imaging to evaluate the dosimetric characteristics and diagnostic efficacy of 68Ga‐FAP‐RGD.	NO	PHASE1|PHASE2	5/1/2022	5/1/2024
NCT05976607	Clinical Study of 18F ‐FAPI‐RGD in Renal Tumor	Not yet recruiting	The goal of this observational study is to learn about the value of 18F‐FAPI‐RGD PET/CT imaging in renal tumor. Participants will undergo clinical evaluation and 18F‐FAPI‐RGD PET/CT examination.	NO		2023–08	2024–02
NCT04222543	Imaging of Tumor Microenvironment in Patients With Oropharyngeal Head and Neck Squamous Cell Carcinoma Using RGD PET/CT Imaging	Unknown	Known risk factors inducing squamous cell carcinomas of the head and neck are tobacco and alcohol intake. However, the incidence of human papillomavirus (HPV)‐related oropharyngeal carcinomas is increasing. It is known that HPV+ and HPV‐ tumors have a different reaction to (chemo)radiotherapy. The exact mechanisms underlying these differences is not yet known but might be caused by changes in vascularity. Therefore the vasculature is imaged with the help of a study specific Gallium‐68‐DOTA‐(RGD)2 PET/CT scan and a CT perfusion scan.	NO	PHASE2	11/22/2019	9/1/2023
NCT05543954	68Ga‐FAPI‐RGD PET/CT Imaging in the Lung Cancer Patients	Recruiting	Based on the high expression of specific receptors on the surface of diseased tissues and neovascularization, noninvasive targeted molecular imaging can be used to visualize lesions in vitro by combining specific ligands labeled with short half‐life isotopes. Lung cancer tissues express fibroblast activating protein FAP, and also have high expression of integrin Î ± VÎ^2^3 receptor on the surface of blood vessels. In this study, a novel dual‐target imaging agent 68Ga‐FAPI‐RGD was used for PET/CT imaging of lung cancer.	NO	EARLY_PHASE1	9/3/2022	12/31/2023
NCT02749019	Dual Integrin Î ± vÎ^2^3 and GRPR Targeting PET Imaging in Breast Cancer Patients	Unknown	This is an open‐label positron emission tomography/computed tomography (PET/CT) study to investigate the diagnostic performance and evaluation efficacy of 68Ga‐NOTA‐BBN‐RGD in breast cancer patients. A single dose of 111–148 Mega‐Becquerel (MBq) 68Ga‐NOTA‐BBN‐RGD will be injected intravenously. Visual and semiquantitative method will be used to assess the PET/CT images.	NO	PHASE1	2015–07	
NCT05013086	177Lu‐AB‐3PRGD2 in Patients With Non Small Cell Lung Cancer	Unknown	This is an open‐label, non‐controlled, non‐randomized study to assess the safety and measure image‐based absorbed dose of 177Lu‐AB‐3PRGD2 in patients with non‐small cell lung cancer (NSCLC) who will undergo radioligand therapy using 177Lu‐AB‐3PRGD.	NO	EARLY_PHASE1	10/1/2021	6/1/2023
NCT00565721	A Proof‐of‐concept Study to Assess the Ability of [18F]AH‐111585 PET Imaging to Detect Tumours and Angiogenesis	Completed	This proof‐of‐concept study is designed to assess the ability of \[18F\]AH‐111585 PET imaging to detect tumors and angiogenesis. Up to 30 evaluable subjects are planned to be included at up to two study centers in the United States. Subjects are considered evaluable if they undergo administration of AH‐111585 (18F) Injection, dynamic and static PET imaging, and tumor tissue acquisition. The targeted population is adult subjects at initial diagnosis or recurrence with tumors â‰¥2.5 cm in diameter who are scheduled to undergo resection or biopsy of the tumor as a result of routine clinical treatment. The tumors must belong to one of the following five types: * High‐grade glioma, including glioblastoma multiforme, anaplastic astrocytoma, and anaplastic oligodendroglioma * Lung cancer, including small cell lung cancer and non‐small cell lung cancer * Head and neck (H\&N) tumors, including laryngeal squamous cell carcinoma, well‐differentiated thyroid and oral cavity carcinoma * Sarcoma * Melanoma Safety will be assessed from the rates of adverse events, changes in vital signs, changes in electrocardiogram (ECG) parameters, changes in physical examination findings, and changes in clinical laboratory findings. Efficacy will be assessed as the correlations between parameters derived from the PET images and the reference standards. The reference standards will be immunohistology for Î ± vÎ^2^3 integrins and other biomarkers specific for oncology and angiogenesis and from the standard of care imaging. Measures obtained from optional DCE‐CT imaging may also be used to compare the uptake and retention of \[18F\]AH‐111585 in tumors obtained from the dynamic PET to assess functional status of the vascular system of the tumor.	YES	PHASE2	2007–11	2012–09
NCT01492192	RGD‐PET‐CT in Cancer Angiogenesis	Terminated	The goal of this clinical research study is to look at two new methods of scanning and see whether they can help researchers predict which tumors will respond to drugs that attack tumor blood supply.	NO	PHASE2	2013–05	2015–09
NCT02325349	PET/CT Imaging of Angiogenesis in Lung or Head and Neck Cancers Prior or During Chemotherapy With Antiangiogenic Agents	Terminated	The primary objective of this Phase II study is to evaluate the use of labeled RGD ligand in PET/CT to predict and/or to early assess the efficacy of chemotherapy including an agent with antiangiogenic effect. The predictive value of this approach will be determined by independent assessors on basis of data at the end of the treatment: RECIST 1.1 criteria for CT or MRI, PERCIST criteria for FDG PET/CT, clinical, endoscopic and histological findings.	NO	PHASE2	3/20/2015	9/20/2018
NCT02481726	68Ga‐AlfatideII for the Differential Diagnosis of Lung Cancer and Lung Tuberculosis by PET/CT	Completed	Comparison of 68Ga‐AlfatideII and 18F‐FDG in differential diagnosis effectiveness toward the solitary pulmonary nodules of lung cancer or tuberculosis.	NO	PHASE1|PHASE2	2014–03	
NCT01582516	Safety Study of Replication‐competent Adenovirus (Delta‐24‐rgd) in Patients With Recurrent Glioblastoma	Completed	In the Netherlands a two‐center investigator‐driven phase I/II clinical trial is initiated in June 2010 testing the oncolytic adenovirus Delta24‐RGD to treat glioblastoma patients. The virus is administrated using convection‐enhanced delivery by four catheters as delivery technique, targeting solid tumor as well as infiltrated tumor cells within the peri‐tumoral brain. Patients will be enrolled in cohorts of 3 per dose‐level. The dose levels to be explored are: 10\^7, 10\^8, 10\^9, 10\^10, 3\*10\^10 and 10\^11 viral particles (vp). Once the MTD has been determined, or the study has reached the highest dose cohort, a further 6 or 9 patients will be enrolled at the MTD and evaluated for safety and preliminary signs of efficacy, such that in total at least 12 patients have received the MTD. The primary objective is to determine the safety and tolerability of Delta‐24‐RGD administered by CED to the tumor and the surrounding infiltrated brain in patients with recurrent GBM. Secondary objectives are to determine the progression‐free survival (PFS), overall survival (OS), and tumor response rate in patients with recurring tumors amenable for surgical resection and treated at the MTD. Cerebrospinal fluid as well as brain interstitial fluid by microdialysis next to the routinely collected samples of blood at various timepoints before, during and after virus infusion. Various neurodegenerative biomarkers as well as markers of immune response will be assessed in these samples. Furthermore extensive sampling and PCR analyses will be performed to evaluate distribution and shedding of the virus.	NO	PHASE1|PHASE2	2010–06	2014–12
NCT05543317	68Ga‐FAPI‐RGD PET/CT for Dual Integrin Î ± vÎ^2^3 and FAP‐targeted Imaging in Patients With Various Types of Cancer and Compared With 18F‐FDG	Completed	As a new dual receptor (integrin Î ± vÎ^2^3 and FAP) targeting PET radiotracer, 68Ga‐FAPI‐RGD is promising as an excellent imaging agent applicable to various cancers. In this research, we investigate the safety, biodistribution and radiation dosimetry of 68Ga‐FAPI‐RGD in healthy volunteers. Moreover, we evaluate the potential usefulness of 68Ga‐FAPI‐RGD positron emission tomography/computed tomography (PET/CT) for the diagnosis of primary and metastatic lesions in various types of cancer, and compared with 18F‐FDG PET/CT.	NO	NA	7/1/2022	12/31/2022
NCT02798406	Combination Adenovirus + Pembrolizumab to Trigger Immune Virus Effects	Completed	Glioblastoma (GBM) and gliosarcoma (GS) are the most common and aggressive forms of malignant brain tumor in adults and can be resistant to conventional therapies. The purpose of this Phase II study is to evaluate how well a recurrent glioblastoma or gliosarcoma tumor responds to one injection of DNX‐2401, a genetically modified oncolytic adenovirus, when delivered directly into the tumor followed by the administration of intravenous pembrolizumab (an immune checkpoint inhibitor) given every 3 weeks for up to 2 years or until disease progression. Funding Source‐FDA OOPD	NO	PHASE2	10/6/2016	6/30/2021
NCT02747290	68Ga‐NOTA‐BBN‐RGD PET/CT in Prostate Cancer Patients	Unknown	This is an open‐label positron emission tomography/computed tomography (PET/CT) study to investigate the diagnostic performance and evaluation efficacy of 68Ga‐NOTA‐BBN‐RGD in prostate cancer patients. A single dose of 111–148 Mega‐Becquerel (MBq) 68Ga‐NOTA‐BBN‐RGD will be injected intravenously. Visual and semiquantitative method will be used to assess the PET/CT images.	NO	EARLY_PHASE1	2014–07	
NCT02817945	Dual SSTR2 and Integrin Î ± vÎ^2^3 Targeting PET/CT Imaging	Recruiting	This is an open‐label positron emission tomography/computed tomography (PET/CT) study to investigate the diagnostic performance and evaluate the efficacy of 68Ga‐NOTA‐3PTATE‐RGD in lung cancer patients and neuroendocrine neoplam patients. A single dose of 111–185 Mega‐Becquerel (MBq) 68Ga‐NOTA‐3P‐TATE‐RGD will be injected intravenously. Visual and semiquantitative method will be used to assess the PET/CT images.	NO	EARLY_PHASE1	6/1/2016	12/31/2022
NCT05976620	Clinical Study of 18F‐FAPI‐RGD in Breast Tumors	Not yet recruiting	The goal of this observational study is to learn about the value of 18F‐FAPI‐RGD PET/CT imaging in breast tumors. Participants will undergo clinical evaluation and 18F‐FAPI‐RGD PET/CT examination.	NO		2023–08	2024–02
NCT02490891	Study of the Angiogenesis by PET/CT in Patients With Lymphoma	Unknown	The aim of the study is to measure tumoral angiogenesis modifications by RGD‐K5 PET/CT before and after two cycles of chemotherapy in patients with lymphoma and a large tumoral mass	NO	PHASE2	2015–11	2020–05
NCT02317393	Contribution of the Imaging to the Expression of intÃ©grines Î ± vÎ^2^3 for the Characterization of Residual Masses of Non‐seminoma Tumors at the End of Chemotherapy	Completed	The purpose of this study is to evaluate the contribution of the imaging to the expression of intÃ©grines Î ± vÎ^2^3 for the characterization of the residual masses of non‐seminoma tumors at the end of chemotherapy. The investigators hope that the results of this first stage of the clinical trial come to consolidate the preclinical results obtained by the investigators team to characterizing the interest and the strong contribution of the use of a tracer resting on the expression of Î ± vÎ^2^3 integrine for the diagnosis of simple necrosed mass at the end of the treatment of a non‐seminoma tumor, so allowing to defer a surgery to about 40% of the patients.	NO	PHASE2	2014–12	2020–03
NCT03655977	Radiation Therapy Planning by Multi‐parametric PET/MRI Imaging in Patients With Cervical Cancer	Unknown	The main goal of this project is to evaluate the potential and feasibility of hybrid PET/MRI functional imaging to non‐invasively measure tumor characteristics for radiation therapy planning (RT) for cervical cancer. It will be assessed how the complementary information of tumor characteristics can contributed to better understanding of tumor delineation. Another endpoint of this study is to evaluate a new PET‐tracer (68Ga‐NODAGA‐ E\[c(RGDyK)\]2) enabling imaging of tumor‐angiogenesis.	NO	NA	9/1/2018	9/1/2021
NCT02197169	DNX‐2401 With Interferon Gamma (IFN‐Î^3^) for Recurrent Glioblastoma or Gliosarcoma Brain Tumors	Completed	Glioblastoma (GBM) and gliosarcoma (GS) are the most common and aggressive forms of malignant primary brain tumor in adults and can be resistant to conventional therapies. The purpose of this Phase Ib study is to evaluate how well a recurrent glioblastoma or gliosarcoma tumor responds to one injection of DNX‐2401, a genetically modified, conditionally replicative and oncolytic human‐derived adenovirus. DNX‐2401 is delivered directly into the tumor where it may establish an active infection by replicating in and killing tumor cells.	NO	PHASE1	9/11/2014	3/15/2018
NCT03384511	The Use of 18F‐ALF‐NOTA‐PRGD2 PET/CT Scan to Predict the Efficacy and Adverse Events of Apatinib in Malignancies.	Completed	This is an open‐label, single‐arm study to explore whether 18F‐ALF‐NOTA‐PRGD2 PET/CT scan can predict the efficacy and adverse events of apatinib in patients with malignancies. Integrin Î ± vÎ^2^3 has been shown to play an important role in angiogenesis and up‐regulated obviously in various types of tumor cells and activated endothelial cells. The arginine‐glycine‐aspartic acid (RGD) tripeptide sequence can bind to integrin Î ± vÎ^2^3 with high affinity and specificity. The 18F‐ALF‐NOTA‐PRGD2 will highly combine with Î ± vÎ^2^3, and thus will monitor the antiangiogenic status. In the current study, investigators propose to evaluate the feasibility of 18F‐RGD PET/CT in monitoring efficacy and adverse events of apatinib in malignancies.	NO	PHASE4	9/30/2016	1/28/2018
NCT00805376	DNX‐2401 (Formerly Known as Delta‐24‐RGD‐4C) for Recurrent Malignant Gliomas	Completed	The goal of this clinical research study is to find the highest tolerable dose of DNX‐2401 that can be injected directly into brain tumors and into the surrounding brain tissue where tumor cells can multiply. A second goal is to study how the new drug DNX‐2401 affects brain tumor cells and the body in general.	NO	PHASE1	2009–02	2015–02
NCT00743353	Exploratory, Phase 0 Study of Positron Emission Tomography (PET) Imaging Agent, F‐18 RGD‐K5	Completed	The purpose of this research study is to get information from volunteers without cancer and patients with cancer who have received a new investigational study agent called, “\[F‐18\] RGDK5,” to evaluate biodistribution and dosimetry for the study agent and determine F‐18 RGD‐K5 uptake in angiogenic tumor. the system.	NO	EARLY_PHASE1	2008–08	2009–01
NCT04191460	Fluorescence‐guided Surgery Using cRGD‐ZW800‐1 in Oral Cancer	Recruiting	This is a two‐staged clinical trial to investigate the feasibility of intraoperative fluorescence imaging (FLI) to adequately assess tumor margins in patients with oral cancer using cRGD‐ZW800‐1.	NO	PHASE2	7/12/2022	3/1/2025
NCT01956734	Virus DNX2401 and Temozolomide in Recurrent Glioblastoma	Completed	Phase I trial, unicentric, uncontrolled. Intratumoral injection or intramural (into the resected tumor cavity) of DNX2401 into brain tissue will be followed by up to two 28days cycles of oral temozolomide (TMZ) in schedule of 7 days on/7 days off to evaluate safety of the combination. Completion of two full cycles of TMZ will be dependent upon tolerance and toxicity. The rationale in using the virus with chemotherapy begins with the lessons learned in many clinical trials in glioblastoma (GBM) about both the great difficulty of treating this disease with monotherapy and the limitations of the therapeutic virus. The best clinical results in recent years have been achieved with combinations of multiple therapeutics efforts, including, maximum resection and chemotherapy, immunotherapy, and targeted therapies. There are very strong preclinical data about the synergy of DNX‐2401 and TMZ proposed in our trial design. The dose‐dense schemes of TMZ like the one we will use, have been developed with the aim to saturate o6‐methylguanine‐DNA‐methyltransferase (MGMT). The published results to date have shown reasonable toxicity albeit with modest efficacy’ these schemes are now in Phase III trials. In addition, autophagy triggered by TMZ could help viral replication in the tumor cells 11. The last argument in favor of this virus + TMZ combination is the proved efficacy in killing GBM tumor stem cells. In vitro and animals models have shown this combination is much more effective that any of the treatments alone against GBM stem cells and the tumors derived from them.	NO	PHASE1	2013–09	2017–03
NCT05549024	68Ga‐RM26‐RGD PET/CT Imaging in the GRPR and Î ± vÎ^2^3 Positive Tumor Patients	Recruiting	Based on the high expression of specific receptors on the surface of diseased tissues and neovascularization, noninvasive targeted molecular imaging can be used to visualize lesions in vitro by combining specific ligands labeled with short half‐life isotopes. In this study, a novel dual‐target imaging agent 68Ga‐RM26‐RGD was used for clinical study of tumor PET/CT imaging to further verify its clinical application value.	NO	EARLY_PHASE1	8/16/2022	12/31/2023
NCT04233476	A Study of 99mTc‐3PRGD2 Injection in Lung Cancer Patient	Completed	The study drug Technetium \[99mTc\] Hydrazinonicotinamide PEGylated Bicyclic RGD Peptide Injectionï¼ˆ99mTc‐3PRGD2ï¼‰ of this study is a novel radioactive diagnostic preparation for clinical use as a nuclear medicine molecular probe for tumor SPECT/CT imaging. After 99mTc‐3PRGD2 is injected into the body, it is specifically taken up by integrin receptor‐positive tumor tissue, and the image of tumor tissue can be obtained by SPECT/CT, This can be used for molecular imaging diagnosis and individualized treatment of common tumors. The primary objective of this study was to evaluate the efficacy of 99mTc‐3PRGD2 for the diagnosis of lymph node metastasis in lung tumors. The minor objective was to evaluate the efficacy of 99mTc‐3PRGD2 in the differential diagnosis of benign and malignant lung tumors and the safety of 99mTc‐3PRGD2 in vivo of humans.	NO	PHASE3	10/12/2019	5/8/2021
NCT01447134	RGD‐K5 in Head and Neck Cancer Patients	Unknown	1. Primary endpoint(s): To determine the relationship between the drug distribution and angiogenesis in head and neck cancer patients. 2. Secondary endpoint(s): To expand the safety database of \[F‐18\]RGD‐K5 and to correlate the parameters from the image study to clinical treatment response and prognosis.	NO		2011–06	2014–06
NCT00988936	Efficacy Study of [F‐18]RGD‐K5 Positron Emission Tomography (PET) as a Tool to Monitor Response to an Anti‐angiogenic Drug	COMPLETED	A pilot Phase II study The primary objective for this study is * To explore the usefulness of \[F‐18\]RGD‐K5 PET/CT to predict efficacy or early response to AvastinÂ® (the anti‐angiogenesis drug) plus chemotherapy treatment before the full course of treatment is completed The secondary objectives for this study are: * To continue safety evaluation by collection of safety data from all patients * To gain experience with \[F‐18\]RGD‐K5 PET/CT in order to improve the study design and conduct of future studies Design: An open label, non‐randomized, uncontrolled, single group assignment, pilot efficacy study Duration: Screening visit (3–4 h), pre‐treatment imaging visit of \[F‐18\]RGD‐K5 PET/CT (\ ~ 3–4 h) and the standard \[F‐18\]FDG PET/CT (\ ~ 3–4 h) or diagnostic CT, followed by two \[F‐18\]RGD‐K5 PET/CT scans, one after the second but before the third AvastinÂ® treatment, and one after the fourth but before the fifth AvastinÂ® treatment, and a follow‐up standard \[F‐18\]FDG PET (\ ~ 3–4 h or diagnostic CT. Procedures: Informed consent, collection of demographic information, medical history, blood labs, physical examination, vital signs, ECGs, three sets of \[F‐18\]RGD‐K5 dosing and imaging scans including pretreatment, early mid‐treatment, and later mid‐treatment, concomitant medication collection, adverse event monitoring, and assessment of tumor response to treatment Patients: Approximately forty^38^ patients with non‐squamous non‐small cell lung cancer, metastatic breast cancer, metastatic colon or rectum cancer who will receive chemotherapy plus AvastinÂ®. This allows for approximately 30 evaluable patients to complete this study at approximately four to eight sites internationally	NO	PHASE2	2009–09	2012–05

RGD‐based radiopharmaceuticals are designed to specifically target integrin αvβ3 receptors. These receptors are proteins found on the surface of cells, including cancer cells and activated endothelial cells involved in angiogenesis. The RGD sequence has a specific structure that allows it to bind to these receptors. To enable imaging of tumor angiogenesis, the RGD‐based radiopharmaceuticals are labeled with short half‐life isotopes like ^68^Ga and ^99m^Tc (Table 4). These isotopes emit positrons (in the case of ^68^Ga) or gamma rays (in the case of ^99m^Tc). PET/CT or SPECT/CT imaging techniques are used to detect and capture these emissions. By administering the radiopharmaceuticals to patients and performing PET/CT or SPECT/CT scans, it becomes possible to visualize and quantify the extent and characteristics of tumor angiogenesis. This imaging approach provides valuable information for various aspects of cancer management. First, it aids in cancer diagnosis by providing detailed images that can help identify the presence and location of tumors. It can also assist in staging, which involves determining the extent and spread of the disease. Furthermore, this imaging technique is useful in treatment planning. By visualizing tumor angiogenesis, healthcare professionals can better understand the blood supply to the tumor and make informed decisions regarding treatment strategies. It can help determine the most appropriate treatment approach, such as the use of chemotherapy, radiation therapy, or targeted therapies. Additionally, molecular imaging of tumor angiogenesis using RGD‐based radiopharmaceuticals allows for the monitoring of therapeutic response. By comparing images taken before and after treatment, healthcare professionals can assess the effectiveness of the chosen treatment and make adjustments if necessary. Hence, RGD‐based radiopharmaceuticals, labeled with short half‐life isotopes like ^68^Ga and ^99m^Tc, target integrin αvβ3 receptors. This enables molecular imaging of tumor angiogenesis using PET/CT or SPECT/CT imaging techniques. By visualizing and quantifying the extent and characteristics of tumor angiogenesis, this imaging approach provides valuable information for cancer diagnosis, staging, treatment planning, and monitoring of therapeutic response. It offers insights into the biology and behavior of tumors, aiding in cancer management and treatment decision‐making.

Additionally, conditionally replicative adenoviruses (CRAds) are a type of adenovirus that have been genetically modified to selectively replicate in cancer cells while sparing normal cells. This targeted replication within cancer cells can lead to their destruction and potentially provide a therapeutic benefit. One specific type of CRAd mentioned in Table 4 is Ad5‐Delta 24RGD. This CRAd is designed to infect and replicate within cancer cells that overexpress integrin αvβ3 receptors, which are commonly found on the surface of tumor cells. By targeting these receptors, Ad5‐Delta 24RGD can selectively infect and replicate within cancer cells, leading to their destruction. Another CRAd mentioned is DNX‐2401. This CRAd is being evaluated in clinical trials for the treatment of glioblastoma, a type of aggressive brain cancer. DNX‐2401 is delivered directly into the tumor, either through intratumoral injection or into the resected tumor cavity. Once inside the tumor, DNX‐2401 can replicate and spread, targeting and destroying cancer cells. In addition to glioblastoma, CRAds are also being studied in various types of cancer, including ovarian cancer. Clinical trials are being conducted to evaluate the safety, tolerability, and efficacy of CRAds in these cancer types. These trials aim to determine the appropriate dosing, administration routes, and potential side effects of CRAds in order to assess their therapeutic potential. By selectively targeting cancer cells and replicating within them, CRAds have the potential to provide a targeted and effective treatment approach for various types of cancer. However, it is important to note that clinical trials are still ongoing, and further research is needed to fully understand the safety and efficacy of CRAds in different cancer types.

Apatinib is an antiangiogenic drug that selectively inhibits vascular endothelial growth factor receptor 2 (VEGFR‐2). VEGFR‐2 is a protein that plays a crucial role in angiogenesis, the process by which new blood vessels are formed to supply nutrients and oxygen to tumors.

By specifically targeting and blocking VEGFR‐2, apatinib disrupts the signaling pathway that promotes angiogenesis. This inhibition of angiogenesis can help to starve tumors of their blood supply, potentially slowing down their growth and progression. Clinical studies are currently being conducted to evaluate the efficacy and safety of apatinib in patients with different malignancies. These studies aim to assess the drug's ability to inhibit angiogenesis and its impact on tumor growth and patient outcomes. In the context of molecular imaging, 18F‐ALF‐NOTA‐PRGD2 PET/CT scans are being used in these clinical studies to predict the response to apatinib treatment. PRGD2 is a peptide that specifically binds to integrin αvβ3 receptors, which are overexpressed in tumor‐associated blood vessels. By labeling PRGD2 with the radioactive isotope 18F and performing PET/CT scans, researchers can visualize and quantify the expression of integrin αvβ3 receptors in tumors. These PET/CT scans provide valuable information about the extent and characteristics of tumor angiogenesis, which can help predict the response to apatinib treatment. By assessing the level of integrin αvβ3 receptor expression, researchers can gain insights into the tumor's angiogenic activity and its potential sensitivity to apatinib therapy (Table 4).

## 10 OTHER PEPTIDES THAT CAN COMPETE WITH RGD

The RGD peptide is one of the most commonly used peptides for targeted cancer therapy. The RGD peptide has a high affinity for integrin receptors, which are overexpressed on the surface of many cells. Other peptides can compete with RGD for integrin receptor binding and have been investigated for targeted cancer therapy. examples of peptides that have been investigated for targeted cancer therapy include the iRGD peptide, which can penetrate tumor tissue and enhance drug delivery,^165^, ^166^, ^167^ and the CRGDK homing peptide, which has a high affinity for αvβ3 integrin.^168^, ^169^, ^170^ However, the RGD peptide remains one of the most widely studied and utilized peptides for targeted cancer therapy due to its high affinity for integrin receptors and its ability to selectively target tumor cells (Table 5).

**TABLE 5:** The application of different pedtides in therapies.

Peptide's type	Applications	Explanation
RGD peptide		
	Specificity	RGD peptides have high specificity toward fibronectin receptors and can selectively bind to them.
	Biocompatibility	RGD peptides are biocompatible and do not cause any adverse immune response in the body.
	Tissue regeneration	RGD peptides promote tissue regeneration by stimulating cell growth, differentiation, and migration.
	Wound healing	RGD peptides stimulate angiogenesis and improve wound healing by increasing the supply of oxygen and nutrients to the wound site.
	Anti‐inflammatory	RGD peptides possess anti‐inflammatory properties and can mitigate the inflammatory response in the body.
	Antitumor	RGD peptides inhibit tumor growth and metastasis by preventing the formation of new blood vessels.
	Drug delivery	RGD peptides can be used as a targeted drug delivery system to deliver drugs specifically to the site of interest.
	Imaging	RGD peptides can be used for imaging purposes to detect cancer cells and other abnormalities in the body.
	Safe	RGD peptides are safe and have been extensively studied for their use in various medical applications.
	Noninvasive	RGD peptides are non‐invasive and can be administered topically or orally, making treatment less painful and more convenient.
NGR peptide		
	Tumor‐targeting	NGR peptides specifically bind to overexpressed CD13 or aminopeptidase N on tumor cells, which can help target the delivery of drugs to these cells.
	Low toxicity	NGR peptides have been shown to be safe and well‐tolerated in both animal and human studies.
	Enhanced drug delivery	NGR peptides have been shown to enhance the delivery of a variety of drugs to tumor cells, including chemotherapy agents, radioisotopes, and gene therapy.
	Antiangiogenic effects	NGR peptides have been shown to inhibit angiogenesis or the formation of new blood vessels, which can help prevent tumor growth and metastasis.
	Immunostimulatory effects	NGR peptides have been shown to activate the immune system and induce antitumor immune responses, which can help eradicate tumors and prevent recurrence.
	Increased therapeutic efficacy	NGR peptides can improve the efficacy of various cancer treatments and increase patient survival rates.
iRGD peptide		
	Enhanced tumor penetration	iRGD peptides improve the penetration of therapeutic agents into tumors by enhancing their binding to tumor‐specific receptors and enabling them to diffuse more deeply into the tumor microenvironment.
	Lower toxicity	The improved tumor penetration of iRGD peptides allows for lower doses of therapeutic agents to be used, reducing the risk of toxicity and side effects.
	Increased efficacy	RGD peptides improve the delivery of therapeutic agents to tumor cells, increasing their efficacy in killing cancer cells and reducing the potential for drug resistance.
	Targeted delivery	iRGD peptides specifically bind to tumor cells, allowing for targeted delivery of therapeutic agents to the site of the tumor, reducing the potential for off‐target effects on healthy cells.
	Improved imaging	iRGD peptides can be used in imaging studies to improve the detection and localization of tumors, facilitating more accurate diagnosis and treatment planning.
	Improved prognosis	The increased efficacy of iRGD peptides in delivering therapeutic agents to tumors can potentially improve patient outcomes and prognosis.
CRGDK peptide		
	Anti‐inflammatory properties	CRGDK peptide has been shown to have anti‐inflammatory properties, which can be beneficial in the treatment of a variety of inflammatory diseases.
	Wound healing	CRGDK peptide has also been found to promote wound healing by stimulating angiogenesis and collagen synthesis.
	Bone regeneration	CRGDK peptide has been shown to promote bone regeneration, making it a potential treatment option for conditions such as osteoporosis and bone fractures.
	Neuroprotection	CRGDK peptide has been found to have neuroprotective properties, which can be beneficial in the treatment of neurological disorders such as Alzheimer's disease and Parkinson's disease.
	Anti‐cancer effects	CRGDK peptide has been shown to have anti‐cancer effects by inducing apoptosis (cell death) in cancer cells.
	Cardiovascular health	CRGDK peptide has also been found to have cardio‐protective properties, which can be beneficial in the treatment of cardiovascular diseases such as hypertension and heart failure.

## 11 iRGD PEPTIDE

The iRGD (internalizing RGD) peptide is a modified version of the traditional RGD peptide with an amino acid sequence: CRGDKGPDC. It has been developed to improve tumor targeting and penetration abilities.^165^, ^166^, ^167^ The RGD motif within the iRGD peptide binds to αvβ3 and αvβ5 integrins overexpressed on tumor endothelial cells, facilitating initial accumulation at the tumor site.^39^, ^171^, ^172^, ^173^ Following proteolytic cleavage of the iRGD peptide after binding to integrins, a CendR motif (C‐terminal arginine or lysine residue) is exposed which interacts with neuropilin‐1 (NRP‐1) receptors present on tumor cells and vasculature, leading to enhanced tissue penetration and cellular uptake via receptor‐mediated endocytosis.^174^, ^175^, ^176^, ^177^ Potential applications of iRGD peptides in cancer treatment include conjugating chemotherapy agents or cytotoxic drugs for targeted delivery, coupling with nanoparticles or nanocarriers for efficient drug transport, enhancing gene therapy approaches by conjugating viral vectors or gene‐editing tools and boosting immunotherapy efficacy through targeted delivery of immune checkpoint inhibitors.

## 12 CRGDK PEPTIDE

The CRGDK peptide is a pentapeptide containing an RGD sequence flanked by cysteine residues at both ends, resulting in improved stability due to cyclization via disulfide bond formation. Its applications are similar to those associated with other RGD‐based peptides but may offer better resistance against enzymatic degradation due to its cyclic structure.^168^, ^169^, ^170^ Some potential applications include tumor targeting via selective binding to αvβ3/αvβ5 integrins overexpressed on cancerous tissues, conjugation with chemotherapeutic or cytotoxic agents for targeted treatments, development of nanoparticles or nanocarriers to improve drug delivery and reduce off‐target effects.^178^, ^179^, ^180^

Both iRGD and CRGDK peptides are derived from the RGD peptide family but possess unique properties that can be exploited for various treatment purposes. The iRGD peptide demonstrates improved tumor targeting and penetration abilities via dual receptor binding (integrins and NRP‐1), while the cyclic structure of CRGDK offers enhanced stability against proteolytic degradation. Each peptide has potential applications in targeted chemotherapy, nanoparticle‐based drug delivery systems, gene therapy, and immunotherapy approaches for cancer treatment.

## 13 INTERACTION BETWEEN THE RGD MOTIF AND INTEGRIN

Integrins are heterodimeric proteins composed of α and β subunits. In humans, there are 18 α subunits and 8 β subunits, which can combine to form different integrin receptors. The extracellular domain of integrins contains a ligand‐binding head region, a single‐pass transmembrane domain, and a cytoplasmic tail that interacts with intracellular signaling molecules. The ligand‐binding head region consists of a β‐propeller domain and a βI‐like domain, which together form a binding pocket for the RGD motif. The RGD motif is a short peptide sequence found in various ECM proteins, such as fibronectin, vitronectin, and collagen. It plays a crucial role in cell adhesion and migration by interacting with integrin receptors. The arginine residue in the RGD motif forms a salt bridge with a conserved glutamic acid residue in the integrin receptor, while the glycine and aspartic acid residues contribute to the overall conformation and stability of the motif.^73^, ^150^, ^181^, ^182^, ^183^

The interaction between the RGD motif and integrin receptors is a multistep process. Initially, the RGD motif binds to the ligand‐binding head region of the integrin receptor, primarily through interactions with the α subunit. This binding induces conformational changes in the integrin, leading to the exposure of a high‐affinity state. This conformational change allows the integrin to bind to other ECM proteins, leading to the formation of focal adhesions and initiation of downstream signaling events. The interaction between the RGD motif and integrin receptors triggers various intracellular signaling pathways. These pathways regulate cell survival, proliferation, migration, and differentiation. One of the well‐studied signaling pathways is the focal adhesion kinase (FAK) pathway, which activates downstream signaling molecules, including mitogen‐activated protein kinases (MAPKs) and phosphatidylinositol 3‐kinase (PI3K). These signaling events ultimately regulate cellular processes such as cytoskeletal rearrangement, gene expression, and cell cycle progression. The RGD motif‐integrin interaction is essential for numerous physiological processes, including embryonic development, tissue homeostasis, and immune response. It is involved in cell adhesion and migration during wound healing, angiogenesis, and tissue regeneration. Dysregulation of the RGD motif‐integrin interaction has been implicated in various pathological conditions, including cancer metastasis, fibrosis, and autoimmune diseases. Targeting this interaction has emerged as a potential therapeutic strategy for these diseases.^10^, ^15^, ^25^, ^184^, ^185^, ^186^, ^187^, ^188^

The interaction between the RGD motif and integrin receptors is a complex and highly regulated process that plays a crucial role in cell adhesion, migration, and signaling. Understanding the detailed information about this interaction provides insights into the mechanisms underlying physiological and pathological processes. Further research in this field may lead to the development of novel therapeutic approaches targeting the RGD motif‐integrin interaction for the treatment of various diseases.

## 14 THE RGD PEPTIDE IN CANCER TARGETING

### 14.1 Breast cancer

Breast cancer is a complex illness with several subtypes that have diverse genomic profiles, clinical characteristics, and therapy responses.^189^ Integrins, a kind of cell surface receptor, are important in cell adhesion, migration, and signaling and are frequently dysregulated in breast cancer. The RGD peptide is known to interact with numerous integrins, making it an attractive option for breast cancer‐targeted therapy and diagnostics.^190^, ^191^, ^192^ Understanding the particular integrin expression patterns in various breast cancer subtypes can aid in the development of RGD peptide‐based techniques for more effective and tailored breast cancer therapy.^10^, ^73^, ^193^

### 14.2 Triple‐negative breast cancer (TNBC)

TNBC is distinguished by the lack of expression of the estrogen receptor (ER), progesterone receptor (PR), and human epidermal growth factor receptor 2 (HER2).^194^ It is a dangerous subtype with few therapeutic choices and a dismal prognosis. Integrins αvβ3, αvβ5, and α5β1 are widely overexpressed in TNBC,^195^, ^196^ and their interaction with RGD peptides has been studied for targeted drug delivery, imaging, and treatment.^197^, ^198^, ^199^, ^200^ MDA‐MB‐231 is a breast cancer cell line generated from a patient with triple‐negative/basal‐like breast cancer, which is distinguished by the lack of ER, PR, and HER2 expression and has a poor prognosis. Another breast cancer cell line produced by a patient with triple‐negative/basal‐like breast cancer is MDA‐MB‐468. It has been found that MDA‐MB‐231 and MDA‐MB‐468 cells have integrins αvβ3, αvβ5, and α5β1, which can bind with RGD peptides (Table 6).^201^, ^202^, ^203^, ^204^, ^205^

**TABLE 6:** The probable integrins in different cancers which could interact with RGD peptide.

Cancer	Cell line	Integrin
Breast cancer		
Triple‐negative breast cancer (TNBC)	MDA‐MB‐231 and MDA‐MB‐468	αvβ3, αvβ5, and α5β1
Luminal breast cancer	MCF‐7, and T47D	αvβ3, αvβ5, and α5β1
Lung cancer		
Non‐small cell lung cancer (NSCLC)	A549, H1299, H460, HCC827, PC‐9 and H1975	αvβ3 and αvβ5
Small cell lung cancer (SCLC)	H69	αvβ3 and αvβ5
Cervical cancer		
Squamous cell carcinoma (SCC)	HeLa, and SiHa	αvβ3 and αvβ5
Colorectal cancer (CRC)		
	HCT116, HT‐29, SW620, Caco‐2, LoVo, LS174T, DLD‐1, SW480 and Colo205	αvβ3
	RKO	αvβ5
Liver cancer		
Hepatocellular carcinoma (HCC)	HepG2 and Huh7	αvβ3, αvβ5, and α5β1
Pancreatic cancer		
Pancreatic ductal adenocarcinoma (PDAC)	PANC‐1, MIA PaCa‐2, BxPC‐3 and AsPC‐1	αvβ3, αvβ5, and α5β1
Renal cell carcinoma (RCC)		
Clear cell RCC (ccRCC)	786‐O and A498	αvβ3, αvβ5, and α5β1

### 14.3 Luminal breast cancer

Luminal breast cancer is distinguished by the presence of hormone receptors (ER and/or PR) and has a better prognosis than TNBC and HER2‐positive breast cancer. Integrins αvβ3, αvβ5, and α5β1 can also be overexpressed in luminal breast cancer, and RGD peptide‐based techniques for targeted therapeutics and diagnostics have been investigated.^206^, ^207^, ^208^, ^209^ MCF‐7 is a frequently used breast cancer cell line obtained from a patient with luminal A breast cancer, which is distinguished by the presence of estrogen receptor (ER) and/or progesterone receptor (PR) expression and low levels of human epidermal growth factor receptor 2 (HER2). T47D is another breast cancer cell line generated from a luminal A breast cancer patient. MCF‐7 and T47D cells have been shown to express integrins αvβ5, and α5β1, which can bind RGD peptides.^151^, ^210^, ^211^ MCF‐7 cells are typically employed as a model for low integrin αvβ3 expression, and similarly, T47D cells exhibit a low level of integrin expression.^52^, ^62^, ^212^, ^213^

### 14.4 Lung cancer

Lung cancer is a diverse collection of cancers that may be divided into two types: non‐small cell lung cancer (NSCLC) and small cell lung cancer (SCLC). NSCLC is further classified into three major subtypes: adenocarcinoma, squamous cell carcinoma, and giant cell carcinoma. SCLC is distinguished by its fast development and early metastasis.^214^, ^215^ RGD peptides have been demonstrated in multiple lung cancer cell lines to bind with integrins αvβ3 and αvβ5.^216^, ^217^, ^218^ These interactions could be used in targeted drug delivery and imaging applications, highlighting the promise of RGD peptides for lung cancer targeted treatment. HCC827 (NSCLC, adenocarcinoma), PC‐9 (NSCLC, adenocarcinoma), H1975 (NSCLC, adenocarcinoma), and H69 (SCLC) are the most often utilized lung cancer cell lines.^73^, ^219^, ^220^, ^221^ These cells have been shown to express integrins αvβ3 and αvβ5, which can bind to RGD peptides.

### 14.5 Cervical cancer

Cervical cancer is the fourth most frequent cancer in women globally, with HPV infection being the leading risk factor. Squamous cell carcinoma (SCC) and adenocarcinoma (AC) are the two primary histological subtypes of cervical cancer.^222^, ^223^ Several integrins have been shown to interact with RGD peptides, adding to the complexity and heterogeneity of cervical cancer. In cervical cancer, integrins that engage with RGD peptides typically bind to fibronectin (α5β1, αvβ3, and αvβ6) and vitronectin (αvβ3 and αvβ5).^224^, ^225^, ^226^, ^227^ Integrin α5β1 overexpression has been found in several cervical cancer cell lines (e.g., HeLa), where it contributes to cell adhesion and migration via interactions with its natural ligand‐fibronectin with an RGD sequence.^224^, ^225^, ^226^, ^228^ Due to its different binding specificity from the other mentioned integrins, Integrin α2β1 does not directly interact with classical linear/cyclic‐RGD peptides, but studies suggest that it may be involved indirectly through crosstalk/signaling pathways related to cervical cancer progression mediated by other RGD‐binding integrins such as α5β1. These integrins' expression and function differ depending on the stage and severity of cervical cancer.^229^, ^230^, ^231^ The use of RGD‐based inhibitors to target these integrins provides a promising treatment option for cervical cancer, with the potential to enhance patient outcomes and lessen the burden of this deadly illness. RGD peptides, on the other hand, have been demonstrated to interact with integrins αvβ3 and αvβ5, particularly binding to fibronectin in cervical cancer cell lines such as HeLa (SCC) and SiHa (SCC).^217^, ^232^

### 14.6 Colorectal cancer

Colorectal cancer (CRC) is a complex disease with several molecular subgroups that can interact with RGD peptides.^10^, ^233^, ^234^ The interaction of integrins with RGD peptides is important in the development, metastasis, and angiogenesis of colorectal cancer and colon cancer. Several integrins, including αvβ3, αvβ5, αvβ6, α5β1, and α2β1, can interact with RGD peptides in CRC.^234^, ^235^, ^236^, ^237^ Accordingly, targeting these integrins and their interactions with RGD peptides may constitute a viable therapeutic method for the treatment of CRC. Integrin αvβ3 is significantly expressed in CRC and has been linked to tumor growth, angiogenesis, and metastasis. The interaction between αvβ3 and RGD peptides increases cell adhesion, migration, and invasion, which contributes to CRC cell aggression. Inhibiting the αvβ3‐RGD connection has been demonstrated in preclinical models to inhibit tumor development and angiogenesis, indicating that targeting this integrin may be a potential treatment approach for CRC. Another RGD‐binding integrin that is increased in CRC is integrin αvβ5. It has been associated with cell adhesion, migration, and invasion regulation, and with the activation of growth factor signaling pathways such as the VEGF pathway. Targeting the αvβ5‐RGD connection might aid in the inhibition of tumor development and angiogenesis in CRC. Integrin expression and interactions with RGD peptides may change between cell lines and subtypes. HCT116, HT‐29, SW620, Caco‐2, LoVo, LS174T, DLD‐1, SW480, and Colo205 colorectal cancer cell lines contain integrin αvβ3, which can interact with RGD peptides.^234^, ^238^ The RKO cell line has been demonstrated to interact with RGD peptides, which can attach to integrin αvβ5.^239^, ^240^

### 14.7 Liver cancer

Hepatocellular carcinoma (HCC), intrahepatic cholangiocarcinoma (ICC), and combination hepatocellular‐cholangiocarcinoma (cHCC‐CC) are all subtypes of liver cancer. The interaction between integrins and RGD peptides is critical in the development, metastasis, and angiogenesis of liver cancer. Several integrins, including αvβ3, αvβ5, α5β1, and α6β1, have been found to interact with RGD peptides in liver cancer.^208^, ^241^, ^242^, ^243^, ^244^

These integrins have been shown to interact with RGD peptides in several liver cancer cell lines. These interactions contribute to tumor growth, metastasis, angiogenesis, and therapy resistance, making them attractive therapeutic targets for the treatment of liver cancer. Integrins αvβ3, αvβ5, and α5β1 are expressed by HepG2, and Huh7 (well‐established HCC cell lines).^245^, ^246^, ^247^

### 14.8 Pancreatic cancer

Pancreatic cancer is a formidable adversary in the world of oncology, characterized by its aggressive nature, limited treatment options, and poor prognosis. Pancreatic cancer is a very aggressive and diverse disease, with pancreatic ductal adenocarcinoma (PDAC) being the most frequent subtype. The search for innovative approaches to combat this deadly disease has led researchers to explore the application of RGD peptides, which have shown promise in targeting integrins—a group of cell surface receptors that play pivotal roles in tumor growth, invasion, angiogenesis, metastasis, and therapy resistance.^248^ In pancreatic cancer, the dysregulation of integrin expression on cancer cells has been extensively studied. Specifically, integrins αvβ3, αvβ5, and αvβ6 have been implicated in pancreatic cancer progression, making them attractive therapeutic targets.^13^ Several other integrins, including α5β1, and α6β1, may also interact with RGD peptides in pancreatic cancer.^249^, ^250^, ^251^, ^252^ Integrins αvβ3, αvβ5, and α5β1 are expressed by PANC‐1, MIA PaCa‐2, BxPC‐3, and AsPC‐1 (all well‐established PDAC cell lines).^253^, ^254^, ^255^, ^256^, ^257^, ^258^

One promising approach involves utilizing RGD peptides with high binding affinity for integrins, particularly αvβ3, to enhance drug delivery specificity. RGD peptides have been incorporated into liposomal drug carriers, allowing the targeted delivery of anticancer therapeutics directly to pancreatic cancer cells. This modification enhances the tumor specificity of vesicles, increasing the precision of treatment.^13^ RGD‐conjugated albumin nanoparticles have shown promise as delivery vehicles for therapeutic agents in pancreatic cancer. These nanoparticles, by targeting integrin avβ3 receptors on pancreatic cancer cells, enhance drug penetration, improve antitumor efficacy, and inhibit tumor growth and metastasis.^259^ RGD peptides have also been employed in conjunction with gemcitabine, a standard drug for pancreatic cancer treatment, to enhance drug penetration into tumors. The coadministration of GEM and RGD peptides has demonstrated effectiveness in reducing tumor size, particularly in cell line‐based xenografts, emphasizing the potential clinical utility of this approach^260^ In the realm of diagnostic imaging, RGD peptides have been utilized to improve the labeling and uptake of superparamagnetic iron oxide (SPIO) nanoparticles in pancreatic cancer cells. This approach enhances the sensitivity of pancreatic cancer imaging, aiding in early detection and precise evaluation.^261^ Another innovative application of RGD in pancreatic cancer involves using a quantum dots‐RGD probe as a photosensitizer in photodynamic therapy (PDT). This approach inhibits cell proliferation and induces apoptosis in pancreatic carcinoma cells, offering a promising avenue for clinical treatment.^262^

The application of RGD peptide in pancreatic cancer has emerged as a multifaceted approach encompassing targeted drug delivery, tumor‐selective immunotherapies, photodynamic therapy, diagnostic imaging, and more. By targeting integrins, RGD peptides offer a promising avenue to enhance the specificity and efficacy of pancreatic cancer diagnosis and therapy. However, continued research is imperative to fully realize the potential of RGD‐based approaches in combating this challenging disease.

### 14.9 Kidney cancer

Kidney cancer, commonly known as renal cell carcinoma (RCC), is a diverse group of malignancies that develop from renal tubular epithelial cells. RCC is classified into four subtypes: clear cell RCC (ccRCC), papillary RCC (pRCC), chromophobe RCC (chRCC), and collecting duct carcinoma (CDC). Each subtype has different histological characteristics, molecular changes, and clinical consequences.^263^, ^264^

The most prevalent subtype of RCC is ccRCC. It is distinguished by transparent cytoplasm and a well‐defined cell membrane. The deletion of the von Hippel–Lindau (VHL) tumor suppressor gene, which leads to the stability of hypoxia‐inducible factors (HIFs) and the stimulation of angiogenesis, is the most prevalent genetic change in ccRCC. Integrins such as αvβ3, αvβ5, and α5β1 may interact with RGD peptides in ccRCC.^265^, ^266^, ^267^ RGD peptides may reduce cell adhesion, migration, and invasion in ccRCC cell lines such as 786‐O and A498, by targeting these integrins. The second most prevalent kind of RCC is pRCC. The presence of papillary structures bordered by cuboidal or columnar cells marks it. pRCC is classified into two subtypes: Type 1, which has a better prognosis, and Type 2, which is more aggressive and has a worse prognosis.^263^, ^264^ The prognosis for CDC is poor, with a high prevalence of metastasis and resistance to traditional therapy.^263^, ^264^ There is little known about the roles of integrins and RGD peptides in chRCC, and CDC. Integrins such as αvβ3 and αvβ5 may, however, have a role in both pRCC, chRCC and CDC, as they are implicated in cell adhesion, migration, and invasion in other RCC subtypes.^9^, ^268^

## 15 CONCLUSION

Integrins are a kind of cell adhesion molecule that is required for the growth and spread of cancer. By targeting these molecules with RGD peptides, researchers were able to selectively deliver drugs to cancer cells and the tumor vasculature while preventing injury to healthy organs. This technique has recently shown a lot of promise, with RGD‐functionalized drug carriers demonstrating increased efficacy and lower toxicity when compared to traditional chemotherapy. RGD‐functionalized drug carriers have also been shown to improve therapeutic efficacy by increasing medicine absorption and retention inside the tumor microenvironment. Because different cancer types may require different treatments or dosages, the precision of this method allows for the development of individualized treatment strategies. RGD peptides and their conjugates can help detect and track the progression of cancer by serving as imaging agents and improving therapy administration. RGD‐conjugates are expected to become a more important weapon in the fight against cancer as research in this field advances.

## AUTHOR CONTRIBUTIONS

**Hossein Javid:** Conceptualization (equal); data curation (equal); formal analysis (equal); methodology (equal); project administration (equal); writing – original draft (equal). **Mahsa Akbari Oryani:** Data curation (equal); formal analysis (equal); validation (equal); writing – original draft (equal). **Nastaran Rezagholinejad:** Conceptualization (equal); resources (equal); software (equal). **Ali Esparham:** Conceptualization (equal); formal analysis (equal); writing – original draft (equal). **Mahboubeh Tajaldini:** Formal analysis (equal); methodology (equal); software (equal). **Mehdi Karimi‐Shahri:** Project administration (equal); supervision (equal); validation (equal); visualization (equal); writing – review and editing (equal).

## FUNDING INFORMATION

Funding information is not applicable/no funding was received.

## CONFLICT OF INTEREST STATEMENT

The authors declare that they have no conflict of interest.

## ETHICAL APPROVAL

Ethics approval for this type of article (A review) is not applicable.

## CODE AVAILABILITY

Not applicable.

## Data Availability

Data sharing not applicable to this article as no datasets were generated or analyzed during the current study.
